# Prostaglandins Isolated from the Octocoral *Plexaura homomalla*: In Silico and In Vitro Studies Against Different Enzymes of Cancer

**DOI:** 10.3390/md18030141

**Published:** 2020-02-27

**Authors:** Diana Ximena Hurtado, Fabio A. Castellanos, Ericsson Coy-Barrera, Edisson Tello

**Affiliations:** 1 Bioprospecting Research Group, Faculty of Engineering, Maestría en diseño y Gestión de Procesos, Universidad de La Sabana, Campus del Puente del Común, Km. 7, Autopista Norte de Bogotá, Chía, Cundinamarca 250001, Colombia; dianahuva@unisabana.edu.co (D.X.H.); fabio.castellanos@unisabana.edu.co (F.A.C.); 2 Bioorganic Chemistry Laboratory, Facultad de Ciencias Básicas y Aplicadas, Universidad Militar Nueva Granada, Cajicá 250247, Colombia; ericsson.coy@unimilitar.edu.co

**Keywords:** octocorals, prostaglandin, molecular docking, breast and lung cancer, p38-kinase, Src-kinase, topoisomerase IIα

## Abstract

Prostaglandin A_2_-AcMe (**1**) and Prostaglandin A_2_ (**2**) were isolated from the octocoral *Plexaura* *homomalla* and three semisynthetic derivatives (**3**–**5**) were then obtained using a reduction protocol. All compounds were identified through one- and two-dimensional (1D and 2D) nuclear magnetic resonance (NMR) experiments. Additionally, evaluation of in vitro cytotoxic activity against the breast (MDA-MB-213) and lung (A549) cancer cell lines, in combination with enzymatic activity and molecular docking studies with the enzymes p38α-kinase, Src-kinase, and topoisomerase IIα, were carried out for compounds **1**–**5** in order to explore their potential as inhibitors of cancer-related molecular targets. Results showed that prostaglandin A_2_ (**2**) was the most potent compound with an IC_50_ of 16.46 and 25.20 μg/mL against MDA-MB-213 and A549 cell lines, respectively. In addition, this compound also inhibited p38α-kinase in 49% and Src-kinase in 59% at 2.5 μM, whereas topoisomerase IIα was inhibited in 64% at 10 μM. Enzymatic activity was found to be consistent with molecular docking simulations, since compound **2** also showed the lowest docking scores against the topoisomerase IIα and Src-kinase (−8.7 and −8.9 kcal/mol, respectively). Thus, molecular docking led to establish some insights into the predicted binding modes. Results suggest that prostaglandin 2 can be considered as a potential lead for development inhibitors against some enzymes present in cancer processes.

## 1. Introduction

Cancer is a group of diseases that affect any part of the human body due to an excess of malignant cells (called carcinogenic). During 2018, the loss of life due to cancer exceeded 9.5 million, which makes this disease the second cause of death in the world, after cardiovascular diseases [1]. There are different types of cancer that affect the world population, with the highest mortality being lung (1.76 million), colon and rectum (880 thousand), stomach (782 thousand), liver (781 thousand), breast (626 thousand), and esophagus (508 thousand). Currently, there are various treatments, but due to the increased mortality rate, it is necessary to search for new alternatives, such as targeted therapies, which consist of the use of drugs that act specifically against a type of cancer [2]. These drugs block the growth and proliferation of cancer cells by interfering with specific molecules such as enzymes. Many of these drugs have been obtained from marine micro and macro-organisms, as the case of cytarabine (Ara-C), a derivative of spongotimidine obtained from the sponge *Thetya crypta*, as well as Trabectedin (Yondelis®), a tetrahydroisoquinoline alkaloid isolated from the tunicate *Ecteinascidia turbinate*. Both compounds are currently used as drugs against cancer [3]. 

In addition, the marine organisms octocorals are known to produce bioactive metabolites, such as *Plexaura homomalla*, which is known to be a natural source of prostaglandins as 15R-PGA_2_ methyl ester acetate and 15R-PGA_2_ methyl ester [4]. These compounds are present in the human body and are involved in muscles contraction and blood pressure control [5]. Prostaglandins display diverse biological activities. For example, PGE and PGF are also associated with cellular proliferation processes. PGA and PGJ are related with the inhibition of cell proliferation, apparently due the interaction of α,β-unsaturated ketone moieties with enzymes associated with different carcinogenic processes [6]. During the last years, some studies have been carried out with prostaglandins, focused mainly on their potential as anti-inflammatory agents, although some of them have been also oriented towards their activity against cancer. However, due to the high costs of this type of research, in silico studies have been widely used for the discovery and development of new drugs, based on the simulation of putative binding modes within active site of target enzymes through molecular docking [6]. As part of a suitable scope, such simulations can also provide important information regarding pharmacokinetics and pharmacodynamics. Thus, the resulting computational modeling leads to the estimation of the best-docked pose of a test ligand [7,8] as well as some insights into the binding mode through interaction type predictions, within the short-term course of developing new drugs.

There are a few studies using molecular docking to analyze compounds with anticancer activity isolated from octocorals. Most of them are related trials on cytotoxic activity of synthetic derivatives against one or several cancer cells lines but other studies are focused on isolated metabolites from sponges and microalgae. The estimated scores obtained by docking simulations have determined the number of interactions between the atoms of the receptor binding site and those of the ligand. For instance, the study carried out by Hegazy et al. [9] reported the in vitro cytotoxic activity of sardysterol against lung cancer cells line (A549, IC_50_ = 27.3 μM). Through in silico studies by molecular docking and molecular mechanical–generalized Born surface area, they also predicted a binding energy of −47.18 kcal/mol against the epidermal growth factor receptor, which is present in several types of cancer. According to the above-mentioned context, the aim of this work was to isolate, identify, and evaluate the in vitro cytotoxic activity of compounds **1**–**5** obtained from the octocoral *Plexaura homomalla*. Such a purpose was complemented with the enzymatic activity assessment and in silico simulations through molecular docking against p38-α-kinase, Src-kinase, and topoisomerase IIα, which are involved in breast and lung cancer processes, in order to contribute to the search for new biomolecules with potential cytotoxic activity. All the above was conducted under a comparative analysis of the in vitro and in silico results focused on the understanding of the insights into ligand–target binding modes based on both experimental and theoretical results. 

## 2. Results and Discussion

### 2.1. Extraction, Identification, and Structure Elucidation

Octocoral *Plexaura homomalla* was collected from the Colombian Caribbean coast. It was kept frozen until it was extracted with DCM/MeOH (v:v = 1:1). In our previous studies, this extract presented anti-cancer activity against lung (A549) and prostate (PC3) cancer cell lines with IC_50_ of 27.2 and 19.4 μg/L, respectively in MTT assay. This is based on the reduction of tetrazolium to formazan in the mitochondria, so it can be carried out only by living cells, and in this way, the amount of formazan measured is proportional to the number of viable cells in the growth phase. Whereby it was chosen for its chemical study with the aim of isolating the compounds responsible for the activity. Crude extract (3.69 g) was fractionated with a mixture of DCM/H_2_O (v:v = 1:1) and the organic extract (2.40 g) was obtained, which once went to a silica gel column chromatography (0.060–0.043) eluting with n-hexane/EtOAc/MeOH increasing the polarity gradient, to obtain 14 sub-fractions (F1–F14). These sub-fractions were submitted to column chromatography until obtaining the prostaglandin A_2_-AcMe **(1)** as a dark yellow oil and the prostaglandin A_2_ (**2**) as a red oil.

**Figure 1:**
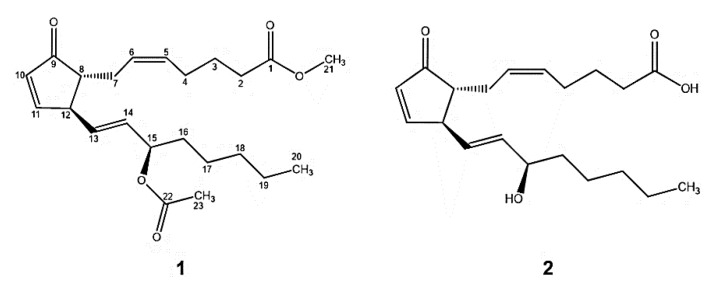
Structures of natural compounds **1** and **2.**

The fraction F5, pure compound **1**, was obtained as a dark yellow oil and the fraction F8, the compound **2** as a red oil. By comparing the signals of its ^1^H-NMR and ^13^C-NMR spectra and its structures (Figure 1), with literature, compound **1** was identified as the (5*Z*,13*E*)-15-acetyl-oxy-9-oxo-prosta-5,10,13-trien-1-oic acid methyl ester a prostaglandin called 15-Ac-PGA_2_-Me, and compound **2** as (5*Z*,13*E*) -15-hydroxy-9-oxoprosta 5,10,13 trien-1-oic acid or commonly known as prostaglandin A_2_ (PGA_2_), previously identified by Reina Gamba [10]. 

### 2.2. Semisynthetic Derivatives

**Figure 2:**
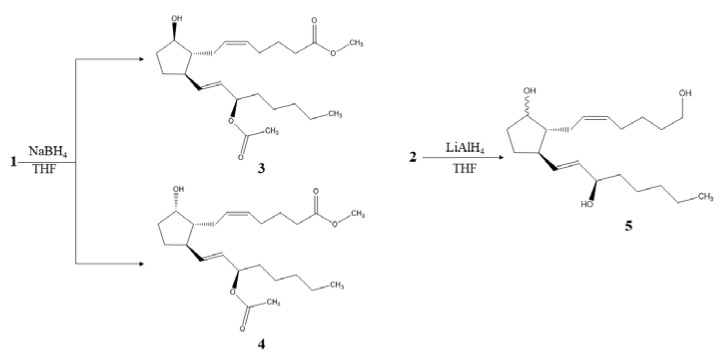
Structures of semisynthetic derivatives **3**–**5.**

The semisynthetic derivatives maintained the prostaglandin skeleton, but some variations in functional groups were found on analyzing their NMR spectra. Figure 2 shows a reduction of **1** using sodium borohydride (NaBH_4_) to obtain the chemical structures of semisynthetic derivatives **3** and **4**. The main change was found in the cyclopentene because the reduction procedure modified both carbonyl and a double bond. In the ^1^H-NMR spectrum (Table 1), a movement of the chemical shift of the hydrogens of C7 were observed due to the hydroxyl group formed, from δ_H_ 2.49 (m)/2.25 (d; *J* = 7.1 Hz) to δ_H_ 2.09 (d; *J* = 7.9 Hz)/2.17 (m) ppm for the derivative **3** and δ_H_ 2.13 (d; *J* = 8.0 Hz)/21.7 (s) ppm for the derivative **4**. In the same way, the hydrogen of C8 resonated to high field from δ_H_ 2.12 (m) to δ_H_ 1.70 (dd; *J* = 14.6, 7.4 Hz) ppm and the hydrogen of C12 from δ_H_ 3.19 (dd; *J* = 7.7, 2.2 Hz) to 2.33 (t; *J* = 7.4 Hz) ppm, for both cases.

Five new signals appear in the ^1^H-NMR spectrum for derivative **3** one in δ_H_ 3.89 (dd; *J* = 11.6, 6.9 Hz) ppm that belongs to the new formed carbinolic methine in C9 and the other four that correspond to the new methylene groups formed by hydrogenation of the double bond appearing in δ_H_ 1.33 (s)/1.59 (d; *J* = 7.5 Hz) and 1.53 (d; *J* = 6.6 Hz)/1.59 (d; *J* = 7.5 Hz) ppm. In addition, in the ^13^C-NMR spectra, the modification of the signal from δ_C_ 210 to δ_C_ 74.8 ppm was observed due to the change of carbonyl to a carbinolic methine. This derivative **3** was identified as (5*Z*,9*β*,13*E*)-15-acetyloxy-9-hydroxy-prosta-5,13-dien-1-oic acid methyl ester known as 15-acetate-11-deoxy-PGF2_β_ methyl ester [11]. As in the previous, for derivative **4** these changes were also observed. However, for C9, the ^1^H and ^13^C-NMR signals were in δ_H_ 4.21 (t; *J* = 4.8 Hz) and δ_C_ 78.1 ppm because the hydroxyl group was located backward, generating steric hindrance. Therefore, derivative **4** was identified as the (5*Z*,9*α*,13*E*)-15-acetyloxy-9-hydroxy-prosta-5,13-dien-1-oic acid methyl ester [12].

The derivative **5** obtained by reduction with lithium aluminum hydride (LiAlH_4_) from the natural compound **2** (Figure 2), showed a reduction of the carbonyl present in the cyclopentene and the carboxylic acid in its respective secondary and primary hydroxyl groups. In Table 1, the data obtained from the ^1^H and ^13^C-NMR spectra can be observed, and the main differences were established, such as the lack of signals corresponding to C1 and C9 quaternary carbons and the presence of two new signals located at δ_C_ 62.9 and 78.5 ppm, corresponding to the carbinolic methylene (C1) and methine (C9), respectively. The ^1^H-NMR spectrum showed the signals at δ_H_ 3.92 (d; *J* = 4.8 Hz) and 4.05 (dd; *J* = 6.4, 3.9 Hz) ppm, which belong to the protons of the methylene (C1) and methine (C9), respectively. These variations, together with the signals located at δ_H_ 1.76 (s) and 1.46 (s)/1.60 (s) ppm, attributed to the methylenes C10 and C11, respectively, allowed to establish this derivative as (5*Z*,13*E*)-1,9,15-triol-prosta-5,13-diene [13].

### 2.3. Cytotoxic Activity

Table 2 shows the results of the cytotoxicity for natural compounds (**1**–**2**) and their semisynthetic derivatives (**3**–**5**) against the human lung cancer cell line (A459) and breast cancer cell line (MDA-MB-231), and two healthy human cells dermal fibroblast cell line (HDFa) and mouse fibroblast cell line (L929).

From the above results, natural compound **1** showed good activity against the breast cancer cells (27.53 μg/mL) but it was ineffective against the lung cancer cells. However, the natural compound **2** had a cytotoxic effect against both types of cancer cells (25.20 μg/mL for lung and 16.46 μg/mL). On the other hand, none of the synthetic derivatives was effective against cancer cells, whereby, the concentration must be greater than 100 μg/mL to inhibit 50% of the cells.

On the other side, it could be established that the derivative **4** instead, of inhibiting the growth of cancer cells, inhibit the growth of healthy cells, which indicates that it could not become a drug in precision medicines because it is not specific for cancer cells. This is the first time that these activities have been reported for this type of prostaglandins because the studies focus on anti-inflammatory activity. However, some studies have been found about cytotoxic effects of prostaglandin D (PGD), which is known as a homologous receptor of chemoattractants and especially PGD_2_-EA (prostaglandin-ethanolamide) and its catabolic product, 15-deoxy-Δ^12,14^-PGJ_2_-EA, which possess cytotoxic activity against skin cancer cells with a IC_50_ of 18 μM and 20 μM respectively, concluding that these are potential therapeutic against skin cancer [14,15]. Showing in this way, that prostaglandins with hydroxyl groups and carboxylic acids have good characteristics as cytotoxic agents and that these can be potentially inhibitory against some types of cancer.

### 2.4. Enzymatic Activity

The signaling pathway of p38 kinase protein activated by mitogens (MAPK) is a part of the family of MAP kinases which phosphorylate the serine and threonine residues, which leads to the regulation of some biological processes such as cell growth, apoptosis, and inflammation [16]. There are four different isoforms, one of them is the p38α MAP kinase that has been characterized for playing an important role in cancer, since a high level of this enzyme has been correlated with breast cancer in patients with highly invasive prognoses, so which is critical for cell migration, invasion, and metastasis. 

**Figure 3:**
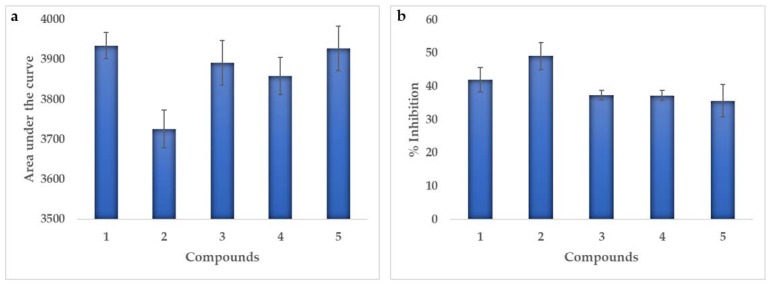
Enzymatic activity for each test compound against the p38α-kinase. (**a**) Area under the curve (AUC), (**b**) Percentage of inhibition at 2.5 μM.

The results did not show sigmoidal behavior, because at high concentrations (10 and 5 μM) the luminescence values were higher, that is, the percentage of inhibition decreased while at concentrations below 2.5 μM a normal behavior was observed increasing the percentage of inhibition as the concentration increased. Possibly due to a higher concentration of compounds that generate interaction with a site of the enzymes different from the active site, which leads to a lower inhibition, as explained by Abdel-Magid [17], where it is stated that most of the drugs are designed to join the primary active sites of the enzymes, known as orthostatic sites. However, these can also have secondary sites or allosteric, in which, at the time of the inhibitors join them, a secondary effect is generated causing an increase in enzymatic activity. Therefore, it was not possible to obtain an IC_50_ value and the results were analyzed by the area under the curve (AUC). This performs the integral of the function of the logarithm of the concentration vs. luminescence for each of the compounds, and in this way to be able to compare the activity between them. The compound that most inhibits the enzymatic activity is one whose area under the curve is smaller, as can be seen in Figure 3a.

All the areas were between 3700 and 4000, observing that the smaller area was in the compound **2**, followed by the derivatives **4** and **3**, which means that the greater inhibition on the enzyme p38α-kinase gives for the compound **2**. In addition, it was also observed that at a concentration of 2.5 μM this compound generated an inhibition of 49%, while the compound **1** generated a 42% inhibition as can be seen in Figure 3b. However, it was also possible to observe that the derivatives inhibition values are less than 40% at this concentration.

On the other hand, there are several enzymes that may be involved in several types of cancer, such as non-receptor tyrosine kinase (c-Src) which belongs to the family of kinases and is expressed in all cell types [18], but its activity is related to advanced malignancies and an unfavorable prognoses of human cancers, so it is considered a molecular target for studies of potential inhibitors. As with the previous enzyme, similar behavior was obtained, so the area under the curve of each of the compounds was also calculated in order to know which of them generate greater inhibition.

**Figure 4:**
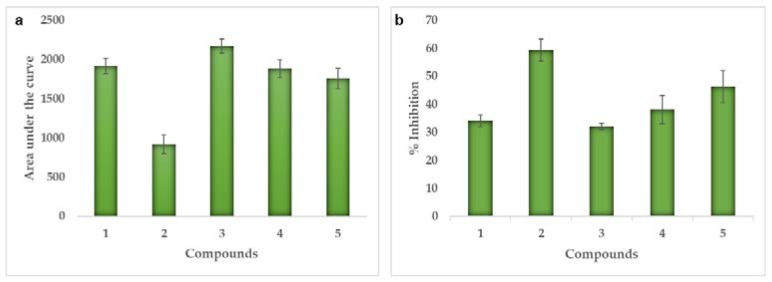
Enzymatic activity for each test compound against Src-kinase. (**a**) Area under the curve (AUC), (**b**) Percentage of inhibition at 2.5 μM.

In Figure 4a, the areas under the curve were observed, which were found between 900 and 2200, showing that the **1** compound and the derivatives had areas above 1500, this suggests that the inhibition of the enzyme is low. While for compound **2** it was 921, that is, the area under the lowest curve. It was corroborated when calculating the inhibition percentage with 59% at a concentration of 2.5 μM, followed by derivative **5** with 46%. For compound **1** and derivatives **3** and **4,** the inhibitions were less than 40%.

**Figure 5:**
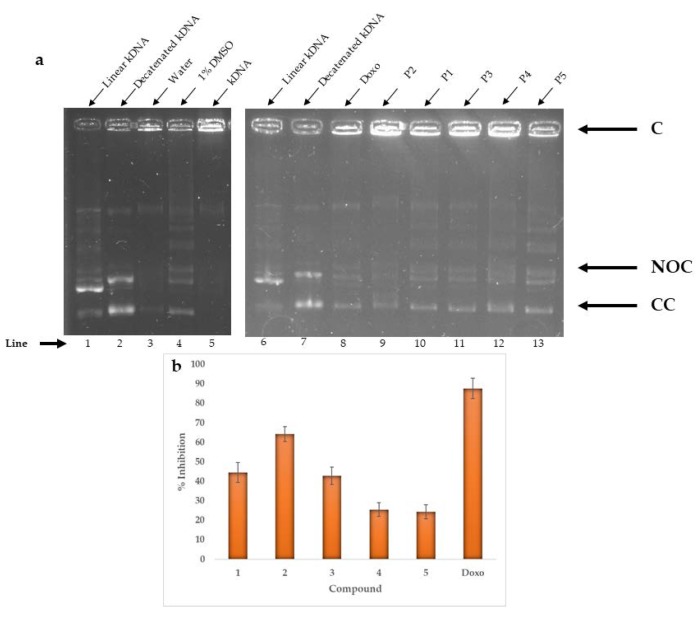
Inhibition of topoisomerase IIα. (**a**) Images of gels for test compounds at 10 μM, (**b**) Percentages of normalized inhibition at 10 μM.

The topoisomerase IIα is a nuclear enzyme that catalyzes the conversion between isomers of DNA and has been observed in a high expression of this enzyme in many types of cancer, including breast cancer and non-small cell lung cancer [19]. Figure 5 shows the 1% agarose gels in which the reaction of the topoisomerase enzyme with compounds was observed and the percentages of inhibition at a concentration of 10 μM. Although it is not clear in a visual way to know which of the compounds generates the greatest inhibition, it was possible to observe that the NOC and CC bands were more intense for the derivatives **4** and **5** than for the rest of the compounds studied. It was also observed that the C band was much more intense for the compound **2** than for the others, which suggests that it is the one that generates the most inhibition. To confirm this information, the percentage of inhibition was calculated by means of normalization with 1% DMSO, showing that doxorubicin (positive control) generated an 88% inhibition at a concentration of 10 μM, while compound **2** obtained 64%, close value to the positive control, for which it could be considered as a potential inhibitor of topoisomerase IIα, present in lung cancer.

### 2.5. Binding Mode Analysis after Molecular Docking Studies

In this study, molecular docking was performed to determine the binding mode (through interactions) and affinity (as Vina scores) of the isolated prostaglandins (**1**–**2**) and their semisynthetic derivatives (**3**–**5**) with p38α-kinase enzyme, using Autodock/Vina. The resulting Vina scores for the best poses of test compounds were observed between −8.5 and −8.0 kcal/mol and revealed at least four intermolecular hydrogen (*H*)-bonds with distances between 2.0 and 2.3 Å (Table S1). Many of these *H*-bonds were formed with particular residues such as Gly_170_ (NH), Leu_171_ (NH), Gly_110_ (NH), Lys_53_ (NH), and Val_158_ (NH and C=O). These observations are in agreement with the study conducted by Abdelhafez et al [20], where the interactions of furocromone and benzofuran derivatives against the same enzyme were studied.

**Figure 6:**
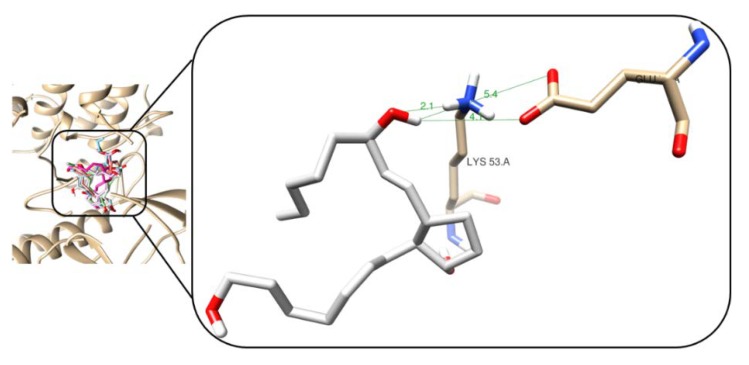
3D interaction models of **5** (white sticks) within the active site of p38α-kinase enzyme (PDB ID: 4FA2). Hydrogen bonds and enzyme residues in dark green lines and light brown sticks, respectively.

The lowest docking score was observed for the derivative **5** with −8.5 kcal/mol. As presented in Figure 6, the 3D interaction model showed that this derivative promoted the formation of one *H*-bond between hydroxyl group at C15 and the terminal NH_3_^+^ of the Lys_53_ side chain. Other two electrostatically weak polar contacts were found between the H of **5** and O of the terminal carboxylate of the Glu_71_ side chain, having distances between 4.1 and 5.4 Å. However, despite compound **2** had a higher amount of polar contacts to that of derivative **5** (as shown in Figure S15), its score resulted in a higher value. This fact can be rationalized due to the presence of weak interactions having longer distances. Two of these electrostatic interactions were established with the peptide bond of some residues, specifically with amide moieties (distances of 5.4 Å), while in the case of **5**, the *H*-bond exhibited a shorter distance with the functional group of Lys_53_ side chains, which appears to promote a stronger stabilization of the enzyme···**5** complex.

Other compounds, such as **1** and **3,** showed the formation of a higher number of weaker interactions and, consequently, higher docking scores. For instance, compound **1** showed *H*-bonds between C=O at C22 and amine (NH) of Gly_110_ and Met_109_, and between C=O of the ring with amine (NH) of Leu_171_ and Gly_170_. However, the scoring function determined a lower ezyme···ligand complex stabilization because the *H*-bonds formed by the protonated amine and carboxyl are stronger to those of *H*-bonds formed with peptide bonds, as observed for compound **1**. Similarly, in the case of derivative **3**, one *H*-bond was observed and its scoring value was also higher. The other three polar interactions were found to be weaker (distances longer than 4.0 Å), which were formed with amide groups of residue peptide bonds. Derivative **4** has only one *H*-bond (2.1 Å).

Table S2 shows the binding scores and *H*-bonds that were formed for each test compound against topoisomerase IIα. Vina scores were found to be in a range between −8.7 and −8.0 kcal/mol. In addition, it was observed that most of the *H*-bonds were formed with Gly_164_, Asn_150_, Asn_120_, Ala_167_, Thr_215_, and Ser_149_ residues, which are part of the enzyme binding site. Similar interactions were observed by Jemimah Naine et al. [21] in their study.

**Figure 7:**
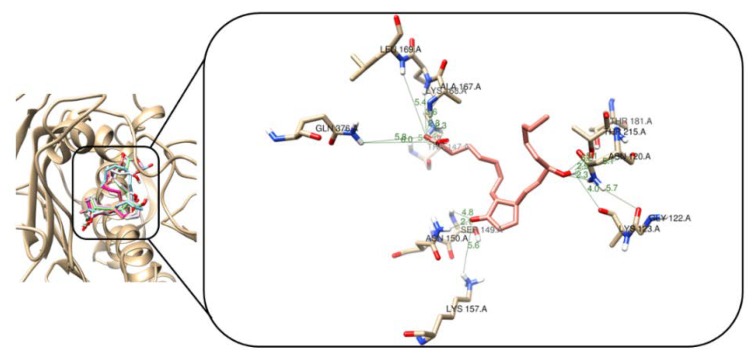
3D interaction models of **2** (light pink sticks) within the active site of topoisomerase IIα enzyme (PDB ID: 1ZXM). Hydrogen bonds and enzyme residues in dark green lines and light brown sticks, respectively.

For topoisomerase IIα, the best interaction profile was achieved for compound **2**, having a Vina score of −8.7 kcal/mol. Four *H*-bonds were observed with distances between 2.1 and 2.3 Å, and other weak polar contacts, as seen in Figure 7. Thus, a *H*-bond was formed between the C=O of cyclopentene and terminal NH_3_^+^ of side chains of Asn_150_ (2.1 Å), other two *H*-bonds were also observed between the NH group of Ala_167_ and the remaining *H*-bond was established with amide carbonyl group of Asn_120_ with hydroxyl oxygen of **2**. Other weaker polar contacts above 3.0 Å were also generated with side chains of particular residues. In the case of the derivative **5**, one *H*-bond was observed (1.8 Å), but other weak polar contacts were formed with residue side chains promoting a highest score among all test compounds. Vina score for compound **1** was found to be higher to that of compound **2**. This fact can be rationalized by the formation of weaker *H*-bonding contacts with particular residues within active site, as exhibited in Figure S16. However, compounds **3** and **4** (derivatives of **1**) generated *H*-bonds with the same active site residues such as Asn_150_, Asn_120_, and Ala_167_ to those of **2**.

**Figure 8:**
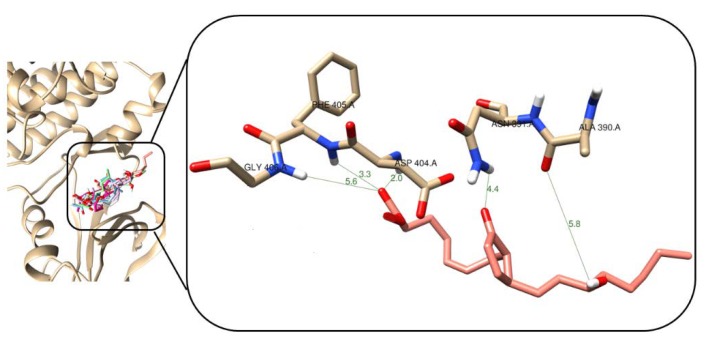
3D interaction models of **2** (light pink sticks) within the active site of Src-kinase enzyme (PDB ID: 2BDF). Hydrogen bonds and enzyme residues in dark green lines and light brown sticks, respectively.

After molecular docking studies with Src-kinase enzyme, it was observed that test compounds formed *H*-bonds with Asp_404_, Glu_310_, Lys_295_, and Ser_345_, which are part of the enzyme active site. Table S3 shows the docking scores for each test compound, which resulted between −8.9 and −7.9 kcal/mol range. The natural compound **2** presented the best interaction profile, having a Vina score of −8.9 kcal/mol and two *H*-bonds with distances between 2.0 and 2.2 Å, as presented in Figure 8. The first of these *H*-bonds was found to be between the oxygen of the carboxyl group and the NH group of the peptide bonds of Asp_404_ (2.0 Å) and the second *H*-bond was observed between the H of the carboxyl group and the carboxylate of the side chain of Glu_310_ (2.2 Å), which can be considered as the most important interaction towards complex stability. In the case of **5** (Figure S17), although it has four *H*-bonds, two of them were created with Asp_404_ and two with Lys_295_, involving a weaker stabilization. In the case of **1** (Figure S17), only an *H*-bond was formed with Asp_404_ within the active site of Src-kinase. For **3** and **4**, an *H*-bond was produced for each resulting complex. In the case of **3,** such an interaction was created with side chain (2.1 Å), while the *H*-bond was originated with amide group for enzyme···**4** complex.

### 2.6. Comparison of In Vitro and In Silico Results

The in vitro experimental results were therefore compared with the in silico results, as presented in Table 3. Compound **2** generated a 50% inhibition of the cell viability of breast cancer (MDA-MB-231) at 16.46 μg/mL but also inhibited the enzyme p38α-kinase by 49% at 2.5 μM. However, molecular docking simulation for the p38α-kinase···**2** complex exhibited the highest Vina score. This difference can be explained due to the fact that p38 has several active sites and the compound **2** may be interacting in a different one from that simulated. In contrast, derivative **5** showed the lowest score (−8.5 kcal/mol), but in vitro inhibited the enzyme by 39% at 2.5 μM. However, in the case of Src-kinase associated with both types of cancer, an inhibition of 59% and binding score of −8.9 kcal/mol were obtained, showing that in silico simulation was confirmed for this enzyme. On the other hand, in the case of lung cancer (A549), associated with the enzymes topoisomerase IIα and Src-kinase, the in silico results fitted adequately with the in vitro results. In this regard, compound **2** inhibited by 50% the cell viability at 25.20 μg/mL, while topoisomerase was inhibited by 64% and the Vina score was −8.7 kcal/mol. For Src-kinase, an experimental inhibition and Vina score of 59% and −8.9 kcal/mol, respectively, were obtained.

Cyclopentenone-containing prostaglandins (such as **1**–**2**) have an enone moiety that eventually has a role as Michael acceptors. This kind of enone moiety can react non-selectively with SH-containing residues (i.e., cysteine) of some molecular targets and proteins, which might be an adequate interpretation of the cytotoxicity observed for compounds **1**–**2**. Therefore, during analysis of the three-dimensional (3D) interaction models after the non-covalent simulation by molecular docking (having a correct convergence of the best pose of the simulation), cysteine residues were looked to be appeared as residual contacts or, at least, near ligands, to deduce that a possible Michael reaction could be favored. However, such proximity was not evidenced in the 3D models. In the future, molecular dynamics simulations could help for tracking such interactions. This can be also rationalized owing to the target proteins of enone-containing prostaglandins are reported to be different to our test enzymes, such as GSH or non-protein thiols [22]. Thus, our purpose to simulate the non-covalent interaction within the active site of this enzyme is particularly relevant to search for these SH-containing residues, on one hand, and to describe some insights into the binding mode of these prostaglandin-type ligands which exhibited inhibitory effect on experimental enzymatic activity, on the other. In addition, the loss of activity when natural compounds are reduced might be explained by other structural reasons to act as electrostatic stabilizers of the enzyme-ligand complex, instead only the enone moiety serving as Michael acceptors. However, a wide chemical space is therefore required to search for and delineate those structural requirements.

In summary, a comparison between in vitro and in silico results was then achieved in the present study. Thus, the molecular docking simulations were validated since in silico results were consistent with the experimental findings, based on the enzymatic activity performed with the same enzymes. This combination of computational and experimental outcome is therefore required to validate and give higher credibility to the in silico studies. Thus, results indicated that compound **2** can be considered as important lead for further studies in cancer research.

## 3. Materials and Methods 

### 3.1. General Experimental Procedures

The ^1^H (400 MHz) and ^13^C (100 MHz) NMR spectra were taken on a Bruker Advance 400 spectrometer (Bruker Corporation, Billerica, MA, USA), using deuterated chloroform (CDCl_3,_ Merck, Darmstadt, Germany); chemical shifts of both the hydrogen and carbon spectrum are found in δ (ppm) and coupling constants (*J*) in Hz. The analytical reactive grade solvents used for the extractions, isolations, and purification of the compounds were hexane (Merck, Darmstadt, Germany), ethyl acetate (EtOAc, Merck, Darmstadt, Germany) and methanol (MeOH, Merck, Darmstadt, Germany). The separation was carried out by column chromatography (CC) with the stationary phase of silica gel 60 (230–450, Alfa Aesar, Switzerland) and silica gel coated plates were used (ALUGRAM® SIL G/UV254, 0.20 mm, MACHEREY-NAGEL, Duren, Germany) for thin-layer chromatography (TLC) analysis.

### 3.2. Animal Material, Extraction, and Separation of Compounds

The octocoral (164 g) was cut into small pieces and extracted with dichloromethane/methanol (DCM/MeOH (v:v = 1:1)) [23,24]. This process was carried out in triplicate, changing the solvent at times 2, 5 and 15 h, then the fractions were grouped and filtered to remove the remaining biological material and obtain a crude extract that was finally concentrated in a vacuum in a rotary evaporator. This process allows obtaining the highest number of metabolites of medium and high polarity present in octocorals.

This crude extract (3.69 g) was fractionated with DCM/H_2_O (v:v = 1:1), an organic extract (2.40 g) was obtained. It was then subjected to gravity chromatography in a silica gel column, eluting with n-hexane/EtOAc/MeOH as the polarity gradient was increased (100% n-hexane to 100% MeOH, v:v). 14 sub-fractions were obtained (F1–F14). A TLC was performed to verify the profile of the compounds in the fractions at different polarities using n-hexane/EtOAc mixture (9:1 and 8:2, v:v). Fraction F5 (512 mg) was subjected to CC using a gradient of n-hexane/EtOAc obtaining 132 sub-fractions each of ~15 mL, which were grouped and concentrated by their profile in TLC to finally give five sub-fractions (F5.1–F5.5). In sub-fraction F5.4, pure compound **1** (402.7 mg) was found, of which a 15 mg sample was solubilized in CDCl_3_ and sent to NMR to obtain its spectra of ^1^H, ^13^C, COZY, HMBC, and HSQC. Later, fraction F8 (923 mg) was also subjected to CC using a gradient of n-hexane/EtOAc obtaining 170 sub-fractions each of ~15 mL, which were grouped and concentrated by their profile in TLC to finally give four sub-fractions (F8.1–F8.4). In sub-fraction F8.3, pure compound **2** (392.7 mg) was found, of which a 15 mg sample was solubilized in CDCl_3_ and sent to NMR to obtains its spectra of ^1^H and ^13^C.

(5*Z*,13*E*)-15-acetyl-oxy-9-oxo-prosta-5,10,13-trien-1-oic acid methyl ester (15-Ac-PGA_2_-Me, **1**): ^1^H-NMR (400 MHz, CDCl_3_): δ_H_ = 7.46 (dd, *J* = 5.7, 2.4 Hz, 1H), 6.17 (dd, *J* = 5.7, 2.1 Hz, 1H), 5.64 (dd, *J* = 15.6, 7.8 Hz, 1H), 5.45 (dd, *J* = 15.6, 6.8 Hz, 1H), 5.39 (d, *J* = 7.1 Hz, 1H), 5.34 (d, *J* = 7.1 Hz, 1H), 5.19 (q, *J* = 6.5 Hz, 1H), 3.65 (s, 3H), 3.19 (dd, *J* = 7.7, 2.2 Hz, 1H), 2.49 (m, 1H), 2.30 (t, *J* = 7.5 Hz, 2H), 2.25 (t, *J* = 7.1 Hz, 1H), 2.12 (m, 1H), 2.07 (q, *J* = 7.2 Hz, 2H), 2.03 (s, 3H), 1.68 (q, *J* = 14.9, 7.5 Hz, 2H), 1.59 (m, 1H), 1.53 (m, 1H), 1.27 (m, 6H), 0.86 (t, *J* = 6.8 Hz, 3H); ^13^C-NMR (100 MHz, CDCl_3_): δ_C_ = 210.4, 174.2, 170.6, 165.0, 133.8, 132.7, 131.4, 130.7, 126.9, 74.4, 52.2, 51.8, 49.8, 34.6, 33.7, 31.8, 27.7, 26.9, 25.1, 25.0, 22.8, 21.6, 14.3

(5*Z*,13*E*) -15-hydroxy-9-oxoprosta 5,10,13 trien-1-oic acid (PGA_2_, **2**): ^1^H-NMR (400 MHz, CDCl_3_): δ_H_ = 7.47 (dd, *J* = 5.7, 2.3 Hz, 1H), 6.18 (dd, *J* = 5.7, 2.0 Hz, 1H), 5.59 (d, *J* = 5.9 Hz, 4H), 5.44 (d, *J* = 10.8, 5.2 Hz, 4H), 4.10 (s, *J* = 3.1 Hz, 1H) 3.22 (d, *J* = 1.9 Hz, 1H), 2.46 (dd, *J* = 13.9, 6.2 Hz, 1H), 2.34 (dd, *J* = 13.2, 6.8 Hz, 3H), 2.13 (m, 3H), 1.70 (m, 2H), 1.52 (d, 2H), 1.30 (s, 6H), 0.89 (s, 3H); ^13^C-NMR (100 MHz, CDCl_3_): δ_C_ = 210.6, 178.2, 165.2, 135.1, 133.5, 131.1, 130.7, 126.9, 72.9, 52.3, 49.7, 37.3, 33.4, 31.8, 26.9, 26.6, 25.2, 24.7, 22.7, 14.1

### 3.3. Semisynthesis of Derivatives

#### 3.3.1. Semi-synthesis of Derivates 3 and 4 from the Compound 1

To a stirred solution of compound **1** (35 mg) in 5 mL of THF, 90 mg of NaBH_4_ was added in two portions. The mixture could stir overnight at room temperature and inert atmosphere, checking the reaction by TLC. The reaction was terminated with 1 mL of 10% NaOH. Then, three washes were carried out with 2 mL of DCM. The organic fractions were grouped and concentrated under vacuum in a rotary evaporator, then subjected to CC using a stationary phase of silica gel and a mixture of h-hexane/EtOAc (9:1 to 3:7, v:v) as mobile phase, to obtain pure derivatives **3** (7.2 mg) and **4** (4.2 mg), which were solubilized in CDCl_3_ and sent to NMR to obtain their spectra of ^1^H and ^13^C.

#### 3.3.2. Semi-synthesis of Derivate 5 from the Compound 2

To a stirred solution of compound **2** 35 mg (1 mol) in 5 mL of THF, 25.7 mg (6 mol) of LiAlH_4_ was added. The mixture could stir for 24 h at room temperature and inert atmosphere, checking the reaction hourly by TLC. The reaction was terminated with 1 mL of NaOH 1M. Then, three washes were made with 5 mL of n-hexane/EtOAc (v:v = 1:1). The organic fractions were grouped and concentrated under vacuum in a rotary evaporator, then subjected to CC using a stationary phase of silica gel and a mixture of n-hexane/EtOAc (7:3 to 2:8, v:v) as mobile phase, to obtain pure derivative **5** (6.3 mg), which was solubilized in CDCl_3_ and sent to NMR to obtain their spectra of ^1^H and ^13^C.

### 3.4. Cytotoxic Activity

All materials and reagents for cell culture were purchased from Sigma Aldrich (Saint Louis, MS, USA). For the assay, two human cancer cell lines and two cell lines, one human and one mouse as controls, were used. The human breast cancer cell line (MDA-MB-231 ATCC®) and lung cancer cell line (A549 ATCC®) were maintained in Dulbecco’s modified Eagle medium (DMEM) supplemented with 10% of fetal bovine serum and 1% of penicillin. The primary human dermal fibroblasts from adult skin (HDFa ATCC®) and the cell line from mouse adipose tissue (L929 ATCC®) were maintained in Roswell Park Memorial Institute medium (RPMI) supplemented with 10% of fetal bovine serum and 1% of penicillin, all cells in incubator with 5% CO_2_ at 37 °C [25].

#### 3.4.1. Cell Proliferation Assay

Antiproliferative studies were carried out using the modified MTT assay based on a previously published method [26]. In this case, 100 μL of cell suspension from the tumor lines was seeded in 96-well plates with a cellular density of 20,000 cells/well and incubated with 5% CO_2_ at 37 °C to allow cell adhesion. After 48 h, the supernatant was discarded and the cells were treated with different concentrations (5, 10, 25, 50, and 100 μg/mL) of the natural compounds **1** and **2** and the semisynthetic derivatives **3**–**5** dissolved in an unsupplemented medium. The plate was incubated for 48 h with 5% CO_2_ at 37 °C. Subsequently, 100 μL of an MTT solution at 0.5 mg/mL was added to each well and placed back in the incubator for 2 h. The supernatant was removed and 100 μL of DMSO was added to incubate for 15 min. Finally, absorbance (OD) at 595 nm was measured on an iMark ™ Microplate reader (Bio-Rad, Hercules, CA, USA).

#### 3.4.2. Anti-Proliferation Quantitative Analysis

The cytotoxic activity was quantified by calculating the mean inhibitory concentration (IC_50_), normalizing the absorbance data and converting them into percentages of inhibition. With a nonlinear 4PL regression, the IC_50_ was found, as developed by Sebaugh [27], using the PrimsGraph Pad® program (GraphPad Software Inc., San Diego, CA, USA).

### 3.5. Enzymatic Activity

#### 3.5.1. Enzymatic Activity from p38α-Kinase and Src-Kinase

For the assay of the enzymatic activity, the p38α Kinase Enzyme System (Promega Corporation, Madison, WI, USA) and Src Kinase Enzyme System (Promega Corporation, Madison, WI, USA) kits were used. In this case 25 μL of reaction were added to each well (in the final concentrations shown in Table 4), where 10 μL were from substrate solution (0.2 mg/mL substrate, ATP and 1 × buffer kinase (Promega Corporation)), 10 μL of kinase solution (kinase (Promega Corporation), 1 × buffer kinase (Promega Corporation) and nuclease-free water) and 5 μL of the compounds initially prepared at 1 mM in DMSO and performing serial dilutions in Buffer Kinase (Promega Corporation) from 10 μM to 0.625 μM according to protocol for the kits (Promega Corporation, 2018). The plates were left at room temperature for 1 h, then 25 μL of ADP-Glo ™ Reagent (Promega Corporation) were added, these were placed at room temperature again for 40 min. Finally, 50 μL of the kinase detection reagent will be added and left at room temperature for 45 min [28]. The measurement was made by means of luminescence in an integration time of 0.5 to 1 s in the GENios Plus microplate reader (Grödig/Salzburg, Austria).

#### 3.5.2. Enzymatic Activity from Topoisomerase IIα

The Topoisomerase IIα drug detection kit (TopoGen, Buena Vista, VA, USA) was used. Reactions were made with 4 μL of reaction buffer (50 mM Tris-HCl, 5 mM ATP, 150 mM NaCl, 30 μg/mL bovine serum albumin, 0.5 mM dithiothreitol, 10 mM MgCl2), 1 μL of the enzyme topoisomerase IIα (supercoiled DNA, in p170 form, 2 units), 1 μL of the kDNA substrate (approximately 200 ng) and 1 μL of the compounds with activity at 10 μM, so that the final volume was 20 μL completing with nuclease-free water. These reactions were incubated at 37 °C for 30 min, and the reaction was terminated with 4 μL of stop buffer. Finally, the solutions were placed in a 1% agarose gel and revealed by electrophoresis for 1 h at 100 V. The results were analyzed by means of the Quantity One program (Bio-Rad, Hercules, CA, USA), where the inhibition percentage was calculated by means of the formula [1- (NOC + CC)_p_/(NOC + CC)_C_]*100, where (NOC + CC)_p_ represent the intensity of the bands for the prostaglandins and (NOC + CC)_C_ the intensity for control bands with 1% DMSO [30].

### 3.6. Molecular Docking

In silico studies were carried out using Autodock Vina 4.2.6 software (The Scripps Research Institute, La Jolla, CA, USA). The X-ray crystal structure of the P38-α-kinase (PDB Code ID: 4FA2), associated with breast cancer, Topoisomerase IIα (PDB Code ID: 1ZXM) associated with lung cancer and Scr kinase (PDB Code ID: 2BDF) associated with both cancers were obtained from the Protein Data Bank. Water molecules were deleted and all missing hydrogen atoms were added based on the protonation state of the protein. The receptor pdbqt file was then prepared according to the AutoDock vina protocol. Finally, the grid center was centered on the binding site at 3 o 4 Å of the flexible residuals, and the grid box size was set to 30 × 30 × 30 points with a grid spacing of 0.375 Å.

The 2D structures of the compound and its synthetic derivatives were constructed by ChemDraw, the 3D structures and minimization of energies were used Chem3D 15.0 (PerkinElmer Inc, Madrid, Spain). Compound **2** was used as a negatively charge molecule through carboxylate moiety. The number of executions of Autodock was set at 50 and the maximum number of energy assessments was set at 2,500,000. Other parameters were set at their default values. For docking, the Lamarckian GA method was used.

## 4. Conclusions

Two compounds were isolated from the octocoral *Plexaura homomalla.* These compounds were identified as Prostaglandin A_2_ type. In addition, three derivatives (**3–5**) were obtained by means of reduction reactions. The Prostaglandin A_2_ (**2**) showed cytotoxic activity against both cancer, breast (MDA-MB-231), and lung (A549) cell lines. On the other hand, it was also possible to establish that the natural compound **2** inhibited p38α-kinase, Src-kinase, and topoisomerase IIα enzymes at different levels. Additionally, molecular docking studies were carried out showing binding scores for all compounds above −8.0 kcal/mol. For the case of p38α-kinase, the best interaction occurred with the derivative **5** forming three *H*-bonds with Lys_53_ and Glu_71_. However, for the other two enzymes, the best interaction was generated with the natural compound **2**. In the case of topoisomerase IIα, 18 *H*-bonds were formed with Asn_150_, Lys_157_, Ser_149_, Thr_147_, Lys_168_, Ala_167_, Leu_169_, Asn_120_, Thr_181_, Thr_215_, Thr_147_, Gln_316_, Gln_122_, and Lys_123_, whereas Src-kinase···**2** complex created six *H*-bonds with Ans_131_, Asp_404_, Phe_405_, Gly_406_, and Ala_390_. On comparing the experimental enzymatic activity and the molecular docking, it was found that the compounds of highest inhibition were also those with lower Vina scores for two of the three test enzymes, suggesting that this tool generates good predictions. Such processing would help to reduce costs and time in further studies of prostaglandin-based inhibitors of such molecular targets related to carcinogenic processes.

## Figures and Tables

**Table 1:** ^1^H and ^13^C NMR spectral data of compounds **3** to **5**.

No.	3		4		5	
	δ_C_	δ_H,_ Multi, (*J* in Hz)	δ_C_	δ_H,_ Multi, (*J* in Hz)	δ_C_	δ_H,_ Multi, (*J* in Hz)
1	175.6	-----	174.1	-----	62.9	3.92 d (4.8)
2	34.5	2.33 t (7.4)	34.4	2.32 t (8.0)	32.3	1.44 s; 1.58 s
3	24.8	1.70 dd (14.6, 7.4)	26.6	1.69 m	25.9	1.55 s
4	26.6	2.09 d (7.9)	29.1	2.09 d, (7.3)	29.9	2.09 q (6.8, 6.3)
5	129.6	5.38 d (7.2)	130.1	5.40 d (7.4)	131,4	5.45 m
6	128.6	5.34 d (7.1)	128.2	5.36 d (7.2)	128.1	5.45 m
7	29.7	2.09 d (7.9); 2.17 s	29.5	2.13 d (8.0); 2.17 s	27.1	2.25 dd (13.9, 4.9); 2.04 s
8	51.7	1.70 dd (14.6, 7.4)	54.1	1,69 m	55.9	1.91 dt (7.5, 3.7)
9	74.8	3.89 dd (11.6, 6.9)	78.1	4.21 t (4.8)	78.5	4.05 dd (6.4, 3.9)
10	33.4	1.53 d (6.6); 1.59 d (7.5)	33.3	1.64 d (9.6); 1.69 m	33.6	1.76 s
11	29.9	1.33 s; 1.59 d (7.5)	29.7	1.33 s	29.3	1.46 s; 1.60 s
12	45.5	2.33 t (7.4)	47.0	2.32 t (8.0)	52.7	2,32 s
13	137.4	5.62 dd (15.3, 8.3)	137.1	5.54 dd (15.3, 8.7)	135.1	5.77 d (20.6)
14	129.3	5.46 dd (13.3, 7.0)	128.7	5.44 dd (11.2, 5.4)	133.2	5.58 dd (15.5, 8.0)
15	73.9	5.19 dd (13.4, 6.7)	74.7	5.19 dd (13.5, 6.8)	72.9	4.05 dd (6.4, 3.9)
16	33.5	1.59 d (7.5); 1.53 d (6.6)	33.4	1.64 d (9.6); 1.69 m	37.5	1.44 s; 1.58 s
17	25.1	1.25 m	24.8	1.25 s	25.3	1.28 s
18	31.5	1.25 m	31.5	1.25 s	31.9	1.28 s
19	22.5	1.25 m	22.5	1.25 s	22.8	1.28 s
20	14.0	0.88 s	14.0	0.88 s	14.2	0.88 s
21	51.5	3.67 s	51.5	3.67 s	-----	-----
22	170.4	-----	170.4	-----	-----	-----
23	21.3	2.04 s	21.4	2.04 s	-----	-----

Recorded in CDCl_3_ and obtained at 400 and 100 MHz for ^1^H and ^13^C NMR, respectively.

**Table 2:** IC_50_ values of the compounds and its derivatives against A549, MDA-MB-231, HDFa, and L929 cell lines

Compound	^1^ IC_50_ (µg/mL)			
	A549	MDA-MB-231	HDFa	L929
1	>100	27.53	28.28	71.74
2	25.20	16.46	17.87	40.39
3	>100	>100	>100	>100
4	>100	>100	50.68	54.33
5	>100	>100	>100	>100

^1^ The IC_50_ values were obtained by adjusting the concentration-response curve to the non-linear regression model in the GraphPad Prism v 8.1 software, San Diego, CA, USA.

**Table 3:** Comparison of in vivo and in silico results.

Compound			1	2	3	4	5
**Cytotoxicity**	MDA-MB-231 (breast cancer)		27.53	16.46	>100	>100	>100
	A549 (lung cancer)		>100	25.20	>100	>100	>100
p38α-Kinase	In vivo (% inhibition)		42	49	37	37	36
	In silico	Scores (kcal/mol)	−8.3	−8.0	−8.1	−8.1	−8.5
		K_i_ (µM)	0.82	1.37	1.16	1.16	0.59
Topoisomerase Iiα	In vivo (% inhibition)		45	64	43	26	24
	In silico	Scores (kcal/mol)	−8.2	−8.7	−8.5	−8.6	−8.0
		K_i_ (µM)	0.98	0.42	0.59	0.50	1.37
Src-Kinase	In vivo (% inhibition)		34	59	32	38	46
	In silico	Scores (kcal/mol)	−8.3	−8.9	−7.9	−8.1	−8.1
		K_i_ (µM)	0.82	0.30	1.62	1.16	1.16

**Table 4:** Substrate and final concentrations of ATP and kinase used in the preparation of solutions [29].

Kinase	Substrate	Concentrations of ATP (µM)	SB10 (ng) ^1^
p38α	p38 substrate	150	4
Src	Src substrate	50	2

^1^ SB10: the amount of kinase needed to generate a 10% conversion of ATP to ADP.

## Supplementary Materials

The following are available online at https://www.mdpi.com/1660-3397/18/3/141/s1, Figures S1–S14: NMR spectrum of compounds. Figures S15–S17: Interactions of compounds with enzymes. Tables S1–S3: Binding energy and feature for the best conformer of each compound in the active site of enzymes.
