# Supplementary material for: Prostaglandins Isolated from the Octocoral Plexaura homomalla: In Silico and In Vitro Studies Against Different Enzymes of Cancer
Source: Mar Drugs. 2020 Feb 27;18(3):141. doi: 10.3390/md18030141 (PMC7143862; doi:10.3390/md18030141)
Supplement: Supplementary file 1 [file marinedrugs-18-00141-s001.pdf]

Article

Prostaglandins Isolated from the Octocoral *Plexaura homomalla*: In Silico and In Vitro Studies Against Different Enzymes of Cancer

Diana Ximena Hurtado [1], Fabio A. Castellanos [1], Ericsson Coy-Barrera [2], and Edisson Tello [1,*]

[1] Bioprospecting research group, Faculty of Engineering, Maestría en diseño y Gestión de Procesos, Universidad de La Sabana, Campus del Puente del Común, Km. 7, Autopista Norte de Bogotá. Chía, Cundinamarca, Colombia

[2] Bioorganic Chemistry Laboratory, Facultad de Ciencias Básicas y Aplicadas, Universidad Militar Nueva Granada, Cajicá 250247, Colombia

* Correspondence: edisson.tello@unisabana.edu.co; Tel.: 571+8615555; Ext: 25219

Index of Figures

Figure S1: ^{1}H NMR spectrum of Ac Prostaglandin A_2 Me **(1)** in $CDCl_3$ 3

Figure S2: ^{13}C NMR spectrum of Ac Prostaglandin A_2 Me **(1)** in $CDCl_3$.......... 4

Figure S3: COSY NMR spectrum of Ac Prostaglandin A_2 Me **(1)** in $CDCl_3$ 5

Figure S4: HSQC NMR spectrum of Ac Prostaglandin A_2 Me **(1)** in $CDCl_3$.......... 6

Figure S5: HMBC NMR spectrum of Ac Prostaglandin A_2 Me **(1)** in $CDCl_3$.......... 7

Figure S6: ^{1}H NMR spectrum of Prostaglandin A_2 **(2)** in $CDCl_3$ 8

Figure S7: ^{13}C NMR spectrum of Prostaglandin A_2 **(2)** in $CDCl_3$ 9

Figure S8: HMBC NMR spectrum of Prostaglandin A_2 **(2)** in $CDCl_3$ 10

Figure S9: ^{1}H NMR spectrum of derivative **3** in $CDCl_3$.......... 11

Figure S10: ^{13}C NMR spectrum of derivative **3** in $CDCl_3$ 12

Figure S11: ^{1}H NMR spectrum of derivative **4** in $CDCl_3$.......... 13

Figure S12: ^{13}C NMR spectrum of derivative **4** in $CDCl_3$ 14

Figure S13: ^{1}H NMR spectrum of derivative **5** in $CDCl_3$.......... 15

Figure S14: ^{13}C NMR spectrum of derivative **5** in $CDCl_3$ 16

Figure S15: 3D interaction models of test compounds within active site of p38α-kinase enzyme (PDB ID: 4FA2) associated with breast cancer. a) **1** (blue sticks); b) **2** (light pink sticks); c) **3** (violet sticks); d) **4** (light green sticks); and e) **5** (white sticks). Hydrogen bonds and enzyme residues in dark green lines and light brown sticks, respectively.......... 17

Figure S16: 3D interaction models of test compounds within active site of topoisomerase IIα enzyme (PDB ID: 1ZXM) associated with lung cancer. a) **1** (blue sticks); b) **2** (light pink sticks); c) **3** (violet sticks); d) **4** (light green sticks); and e) **5** (white sticks). Hydrogen bonds and enzyme residues in dark green lines and light brown sticks, respectively. 18

Figure S17: 3D interaction models of test compounds within active site of Src-kinase enzyme (PDB ID: 2BDF) associated with breast and lung cancer. a) **1** (blue sticks); b) **2** (light pink sticks); c) **3** (violet sticks); d) **4** (light green sticks); and e) **5** (white sticks). Hydrogen bonds and enzyme residues in dark green lines and light brown sticks, respectively. 19

Table S1: Vina scores and binding features for the best pose of each test compound within the active site of p38α-kinase (PDB ID: 4FA2)……20
Table S2: Vina scores and binding features for the best pose of each test compound within the active site of topoisomerase IIα (PDB ID: 1ZXM)……21
Table S3: Vina scores and binding features for the best pose of each test compound within the active site of Src-kinase (PDB ID: 2BDF)……22

Figure S1: ¹H NMR spectrum of Prostaglandin A_2-AcMe **(1)** in $CDCl_3$

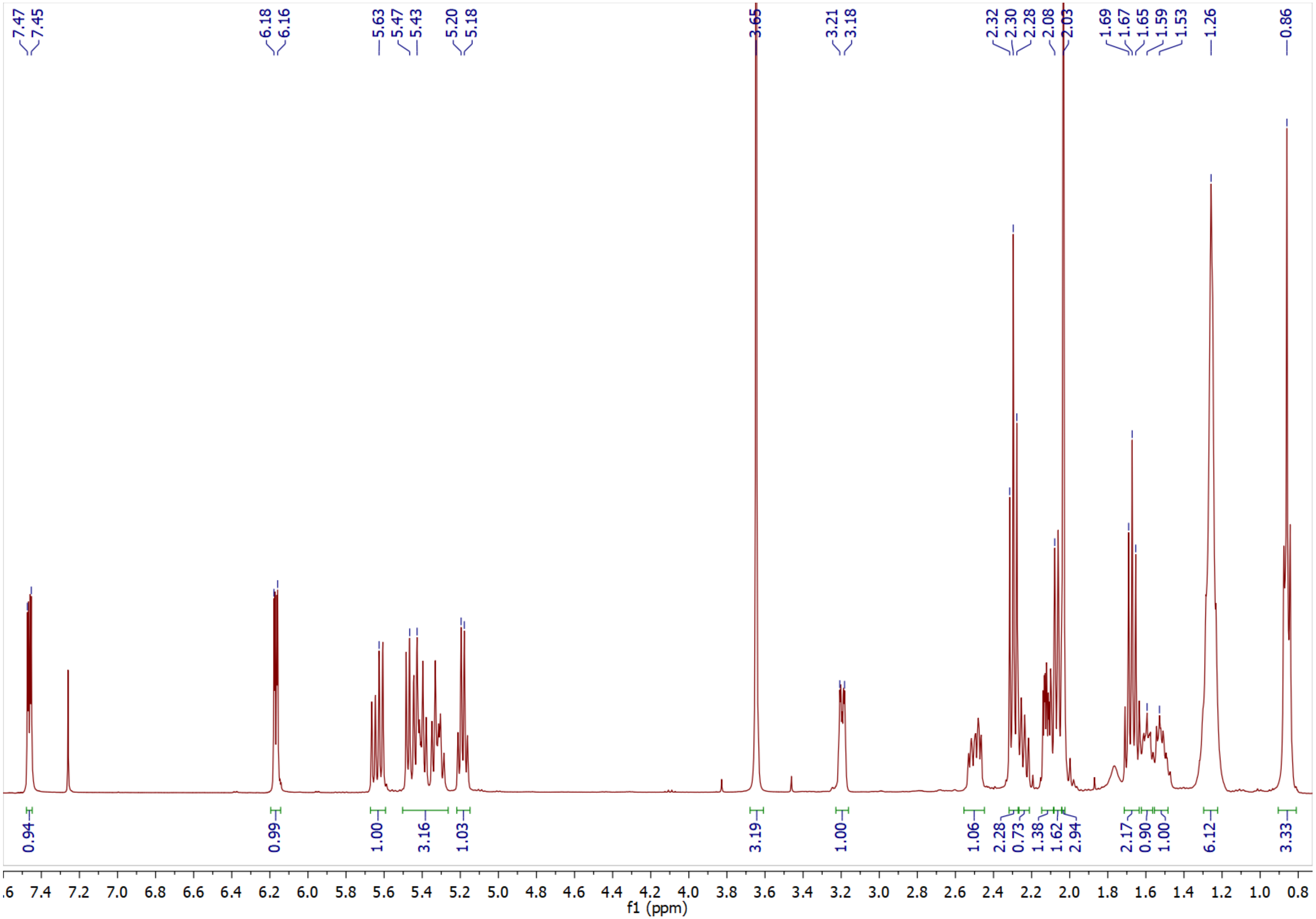

Figure S2: ^{13}C NMR spectrum of Prostaglandin A_2-AcMe **(1)** in $CDCl_3$

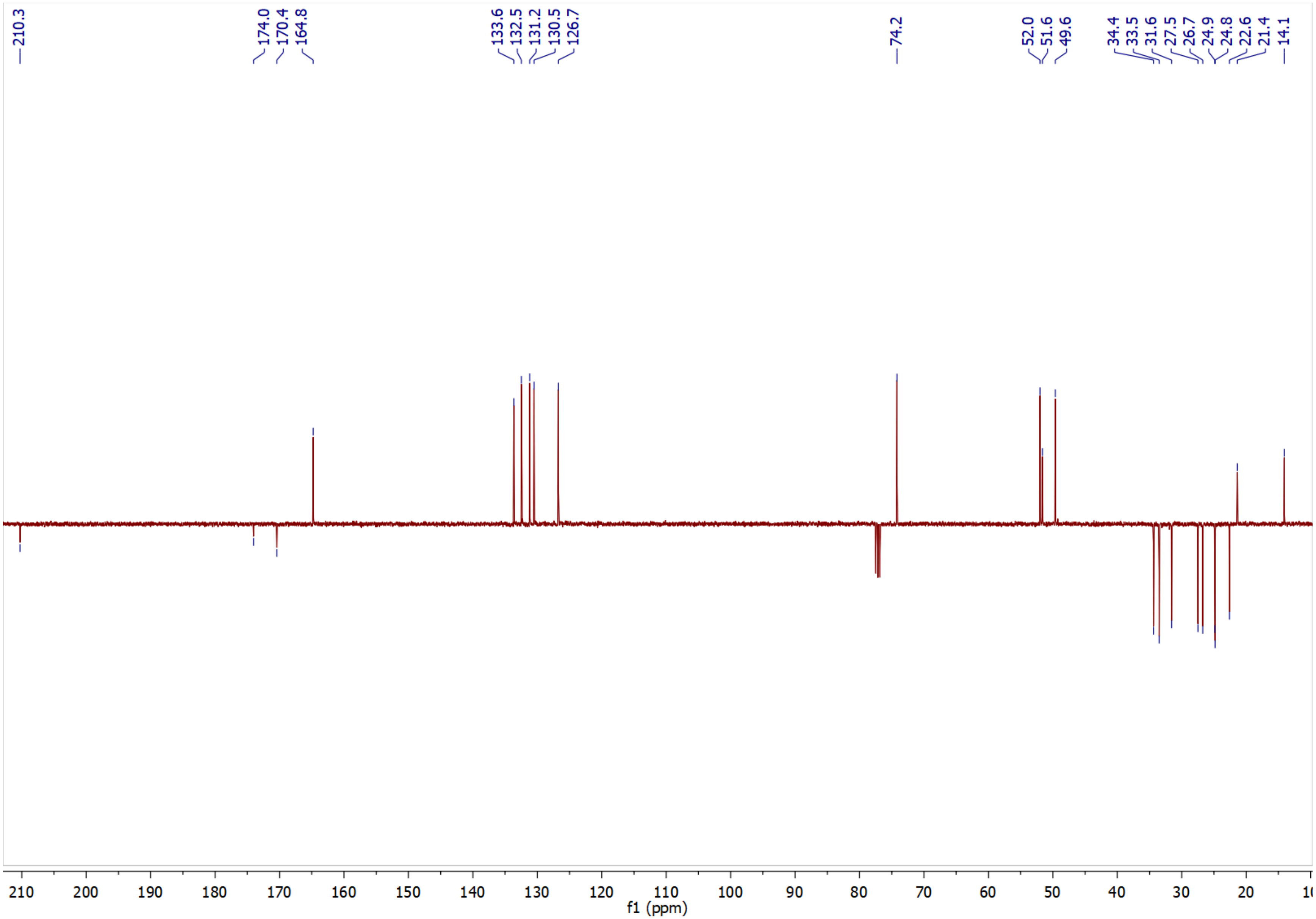

Figure S3: COSY NMR spectrum of Prostaglandin A2-AcMe **(1)** in $CDCl_3$

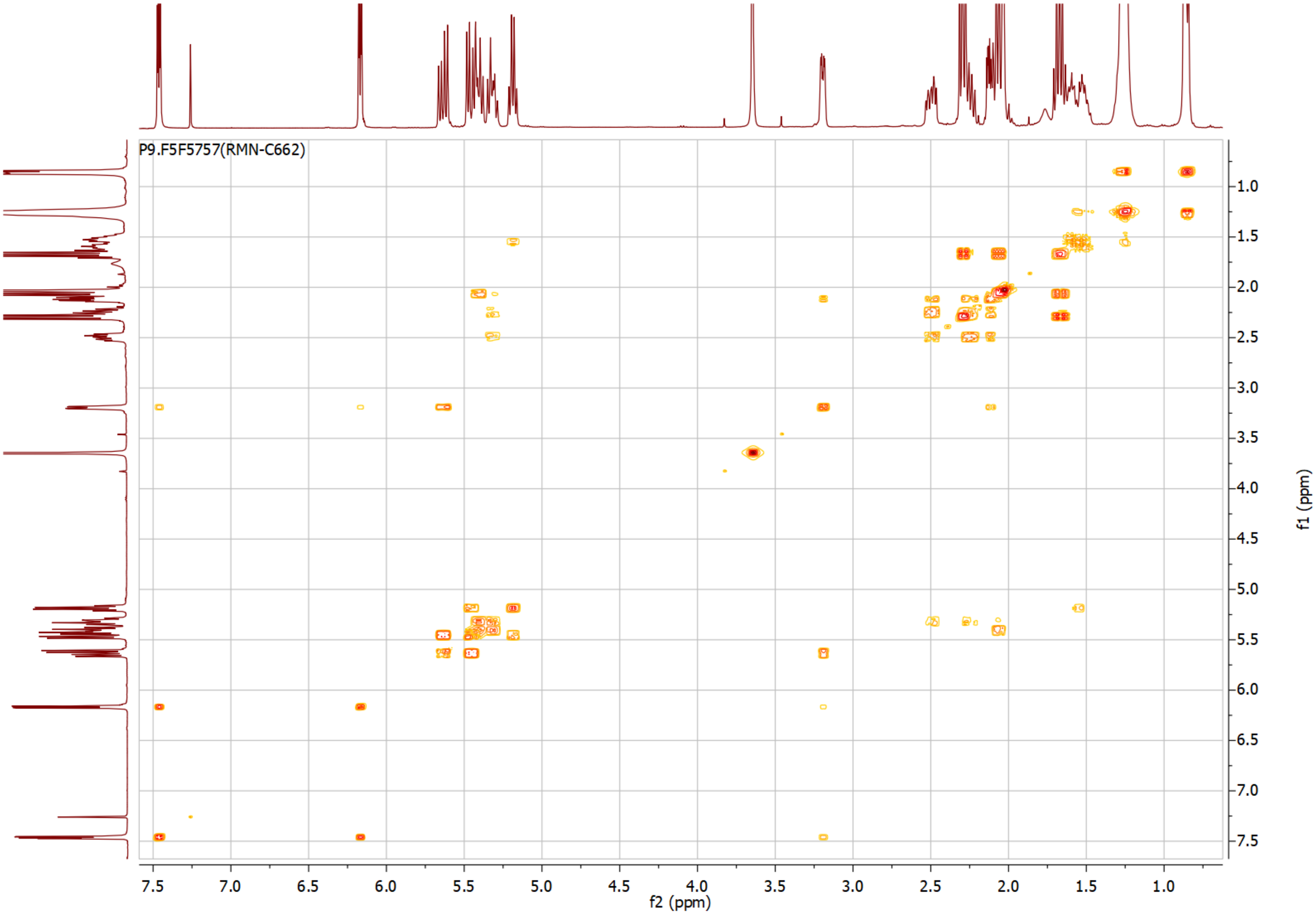

Figure S4: HSQC NMR spectrum of Prostaglandin A_2-AcMe **(1)** in $CDCl_3$

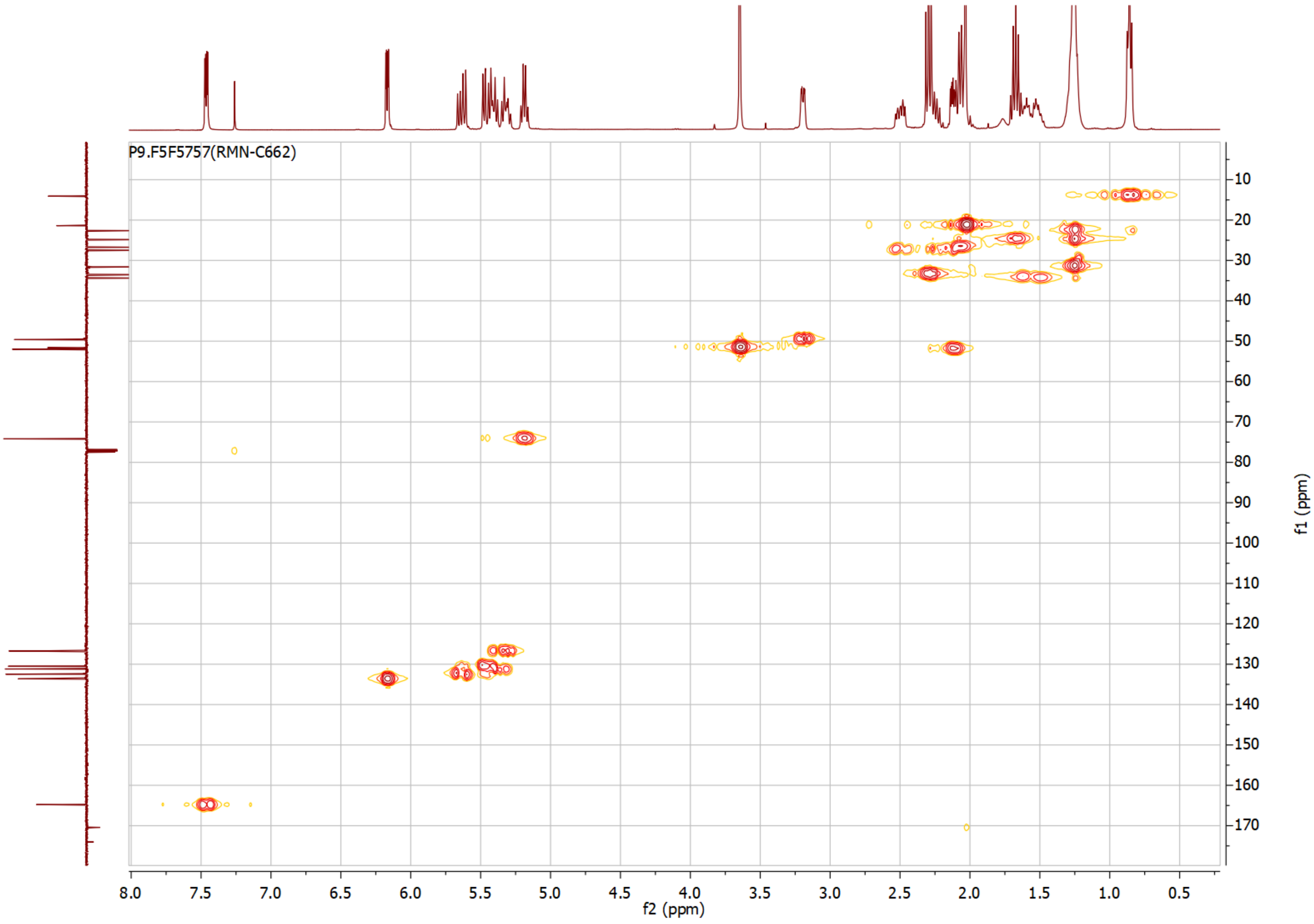

Figure S5: HMBC NMR spectrum of Prostaglandin A2-AcMe **(1)** in $CDCl_3$

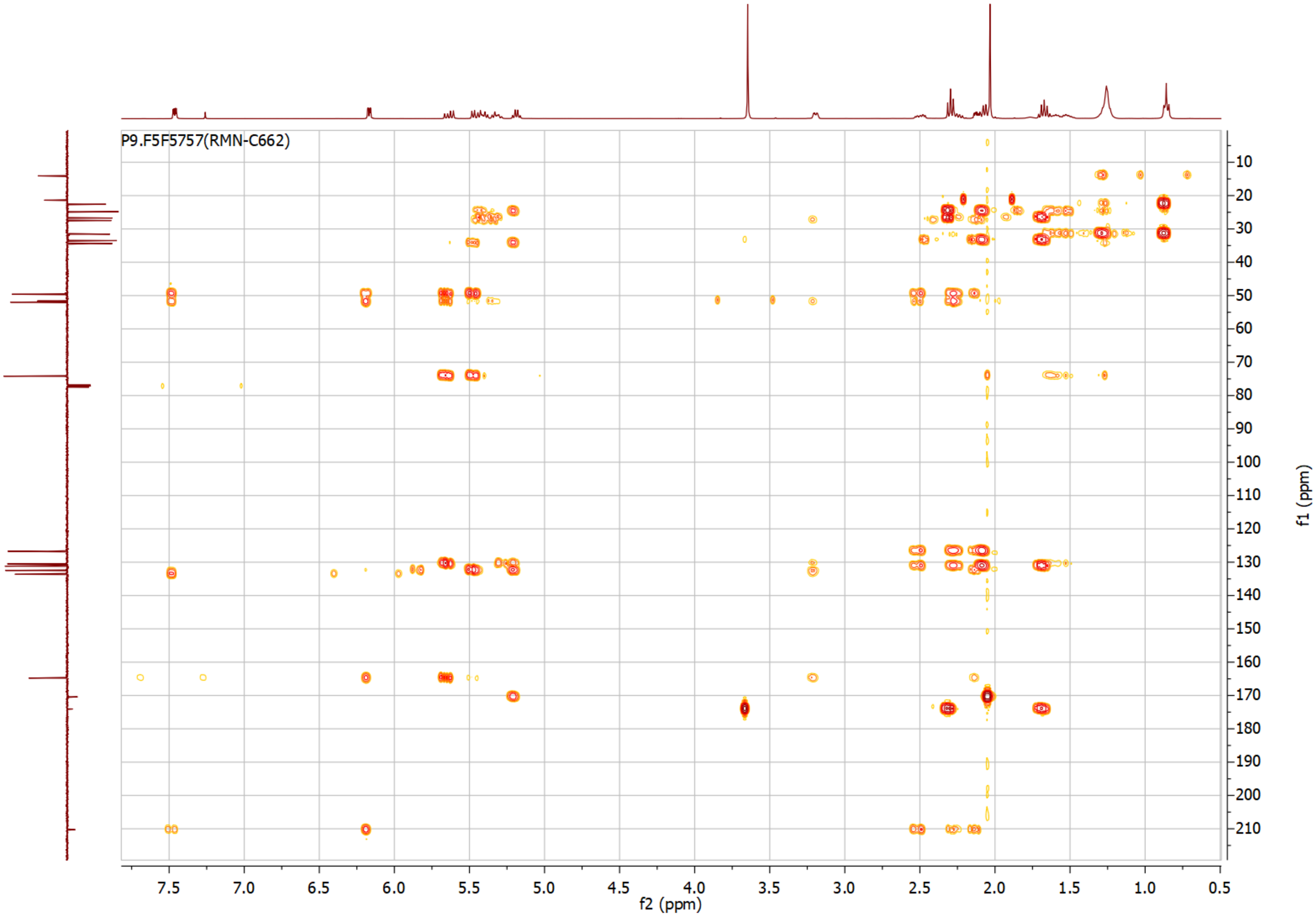

Figure S6: ^{1}H NMR spectrum of Prostaglandin A_2 **(2)** in $CDCl_3$

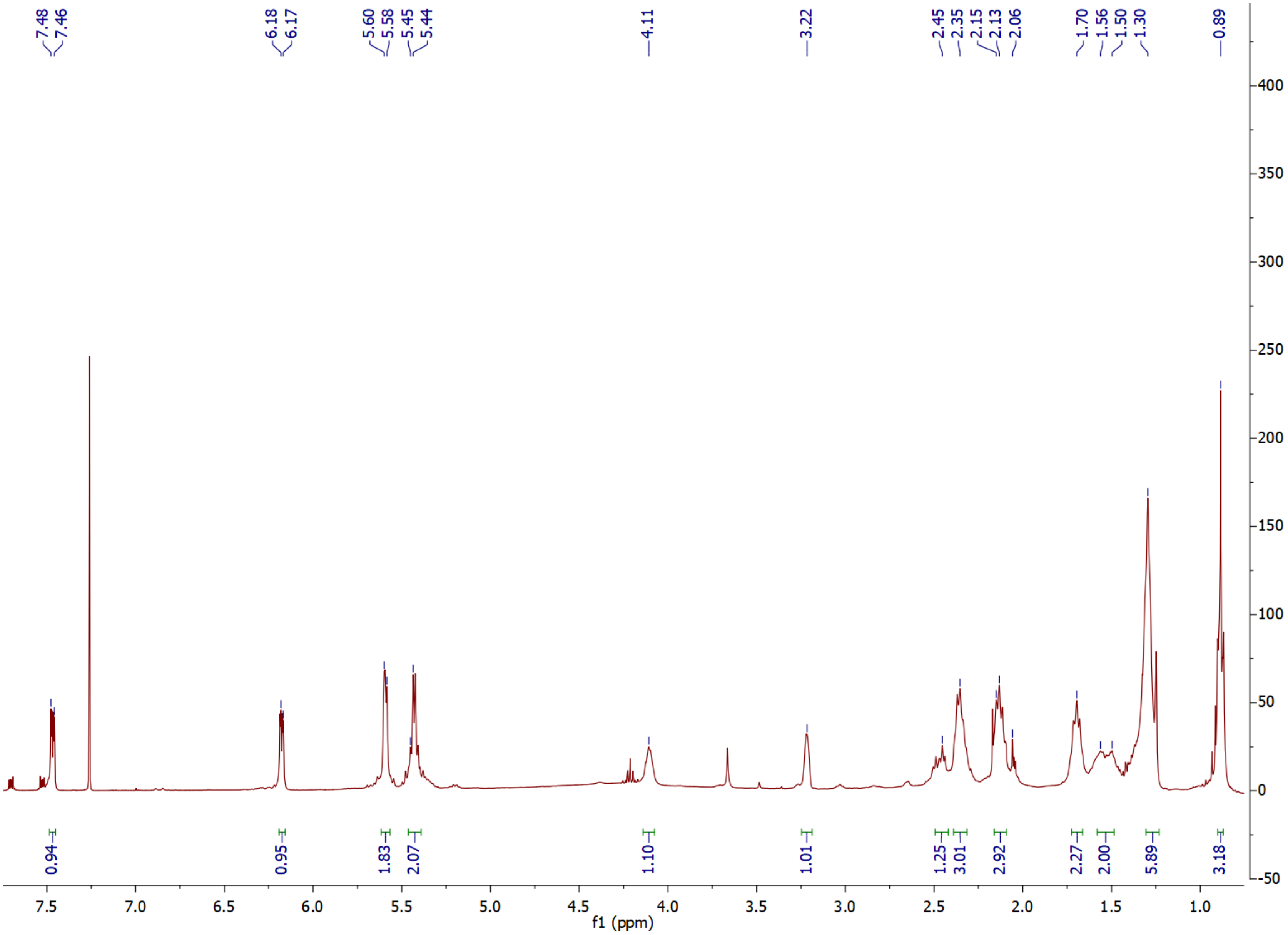

Figure S7: ^{13}C NMR spectrum of Prostaglandin A_2 **(2)** in $CDCl_3$

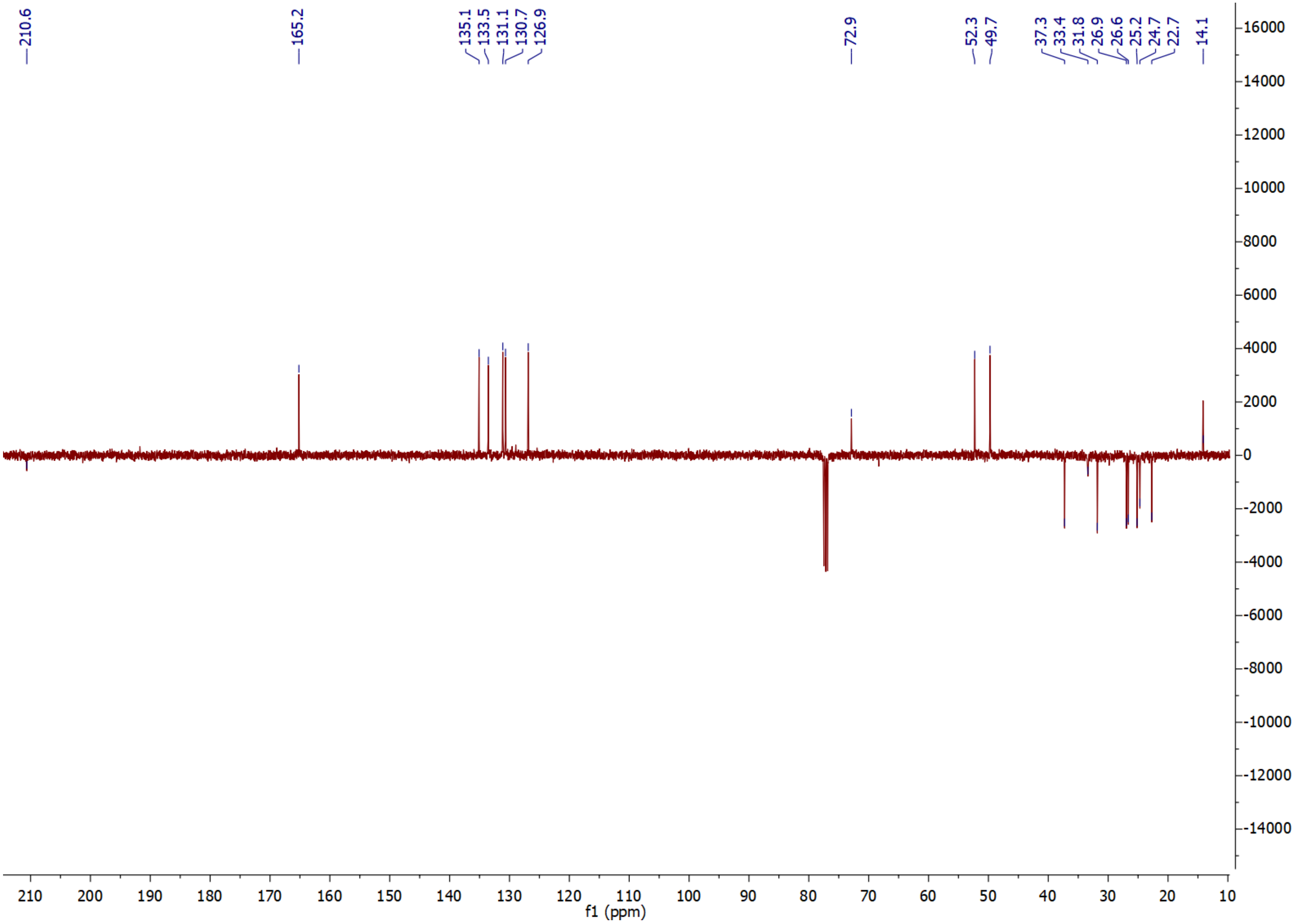

Figure S8: HMBC NMR spectrum of Prostaglandin A_2 **(2)** in $CDCl_3$

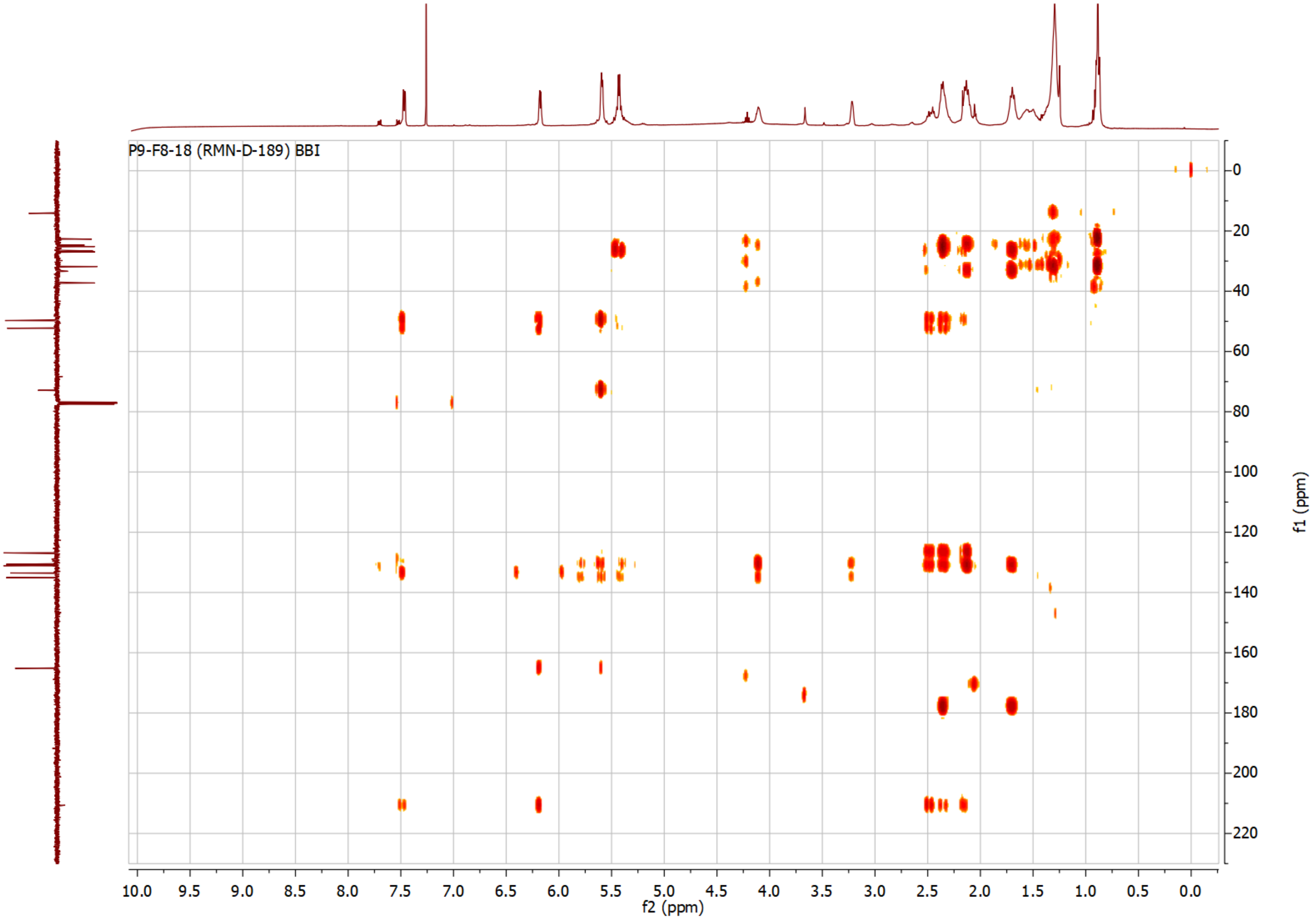

Figure S9: ^{1}H NMR spectrum of derivative **3** in $CDCl_3$

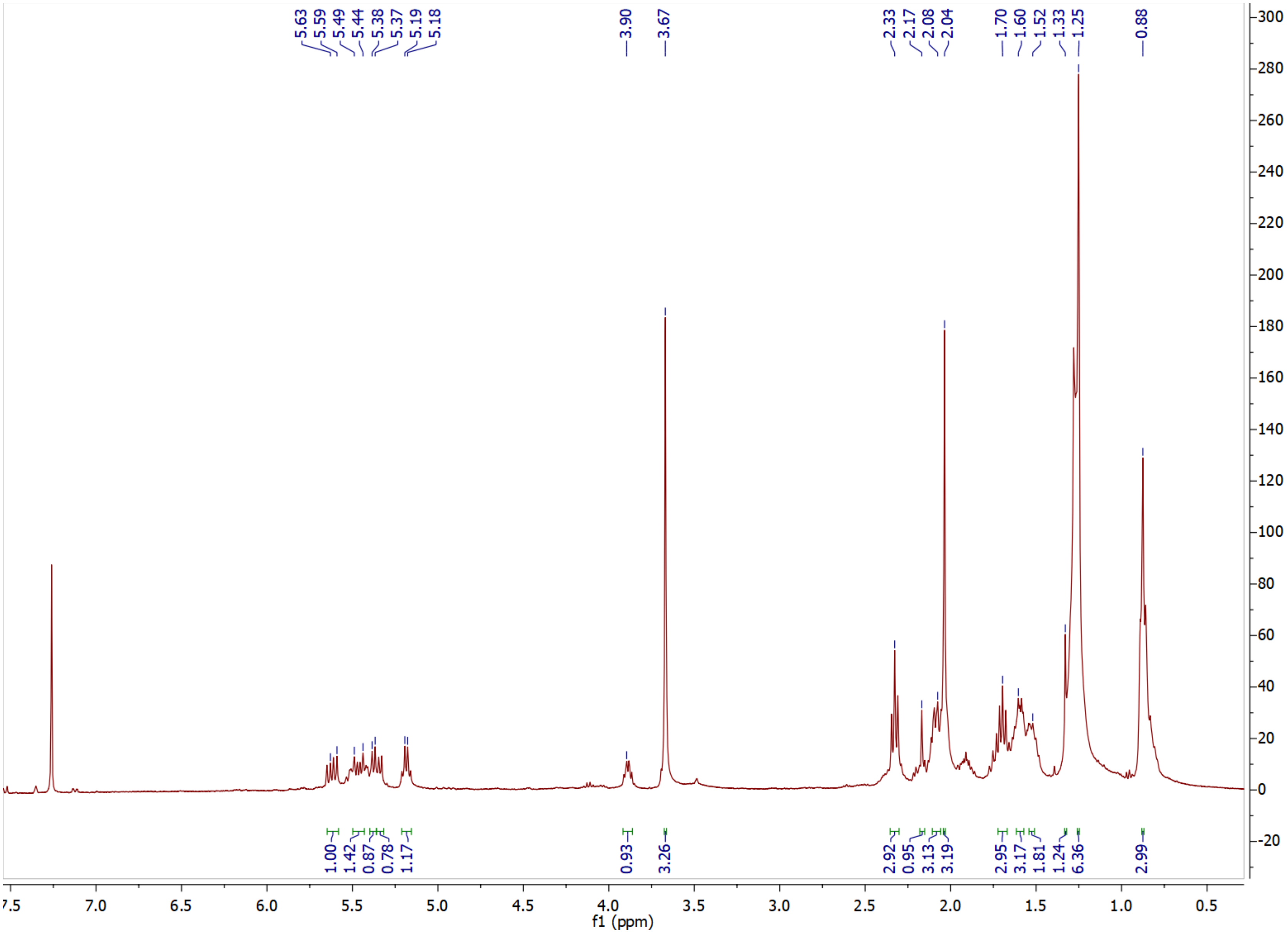

Figure S10: ^{13}C NMR spectrum of derivative **3** in $CDCl_3$

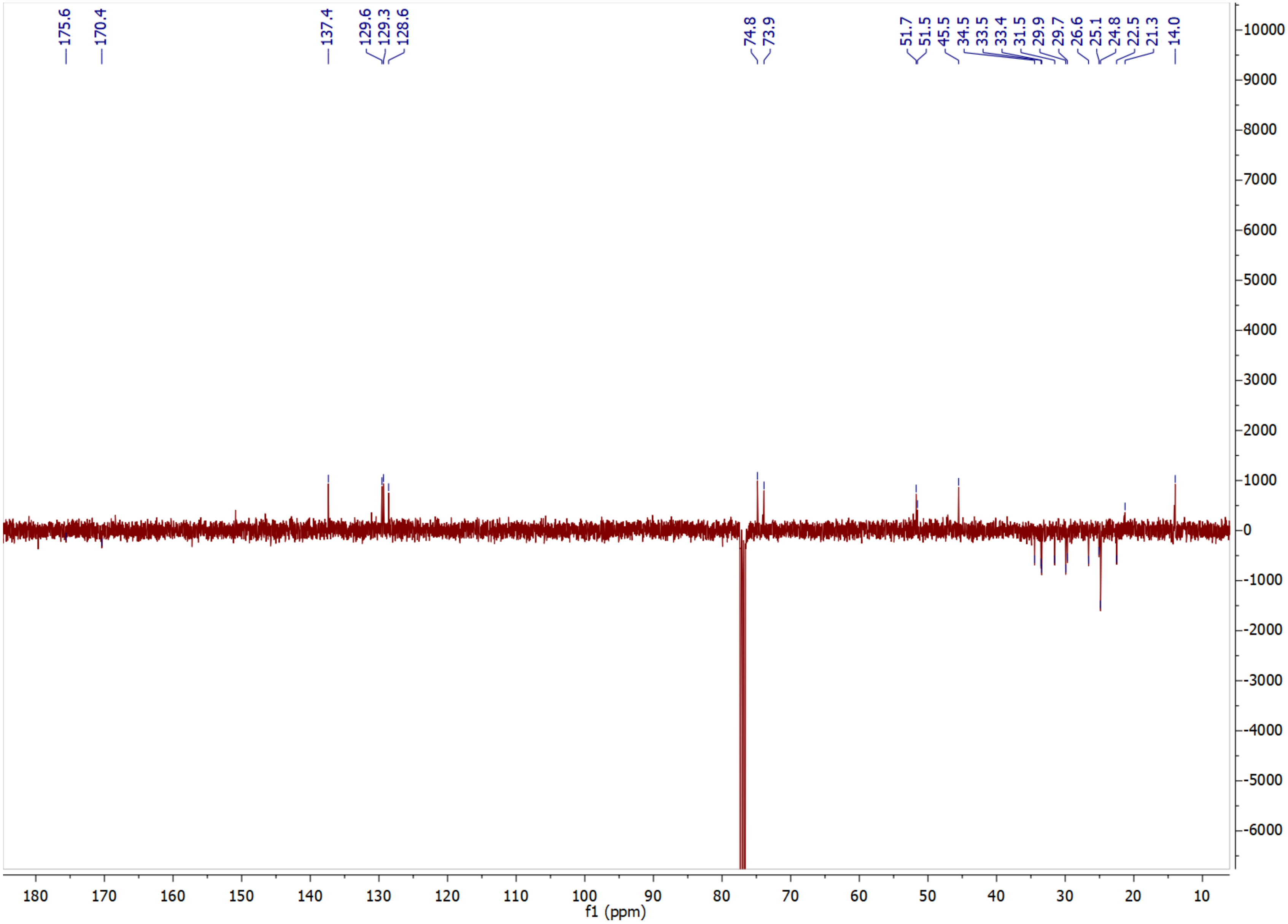

Figure S11: ^{1}H NMR spectrum of derivative **4** in $CDCl_3$

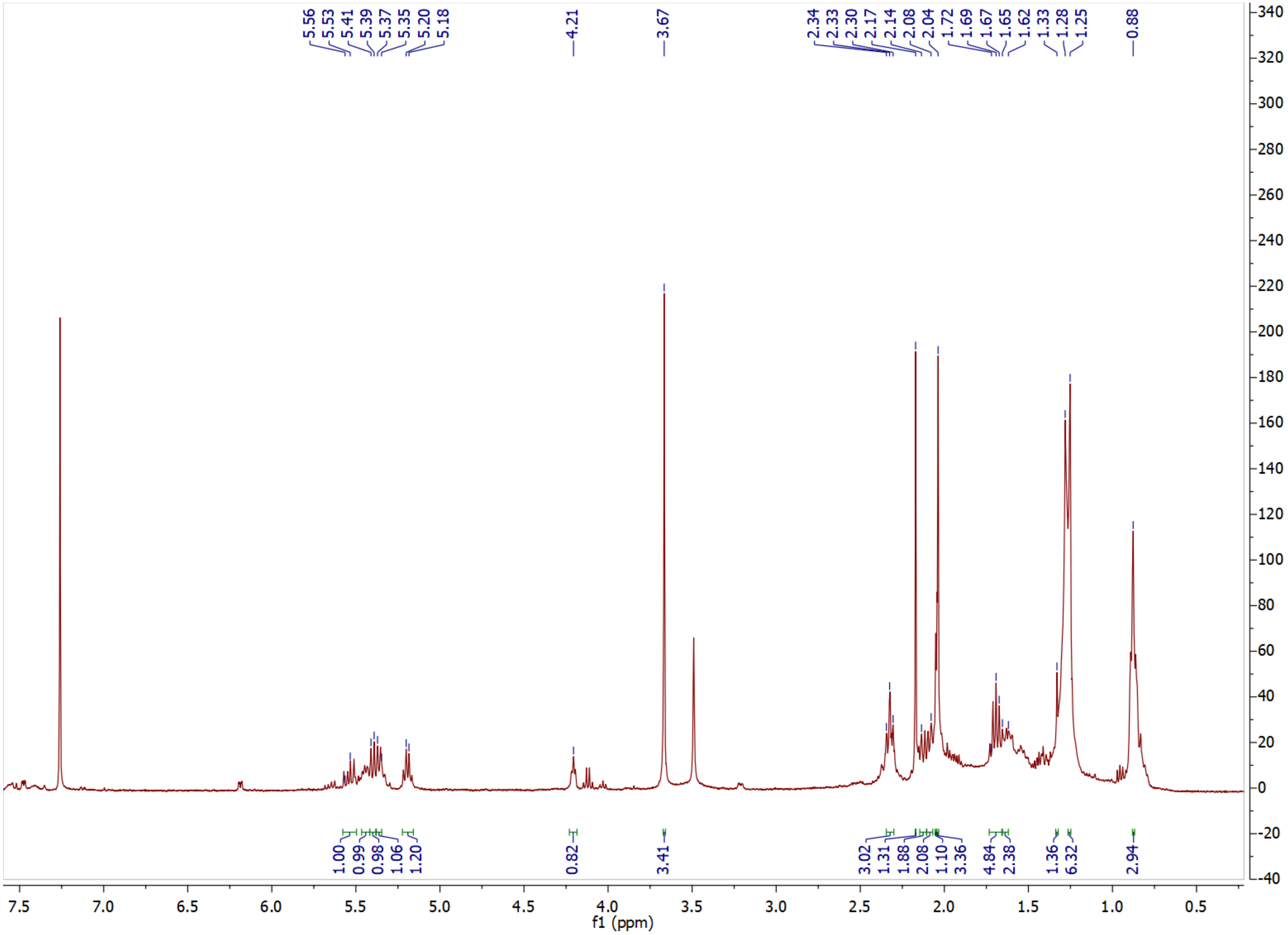

Figure S12: ^{13}C NMR spectrum of derivative **4** in $CDCl_3$

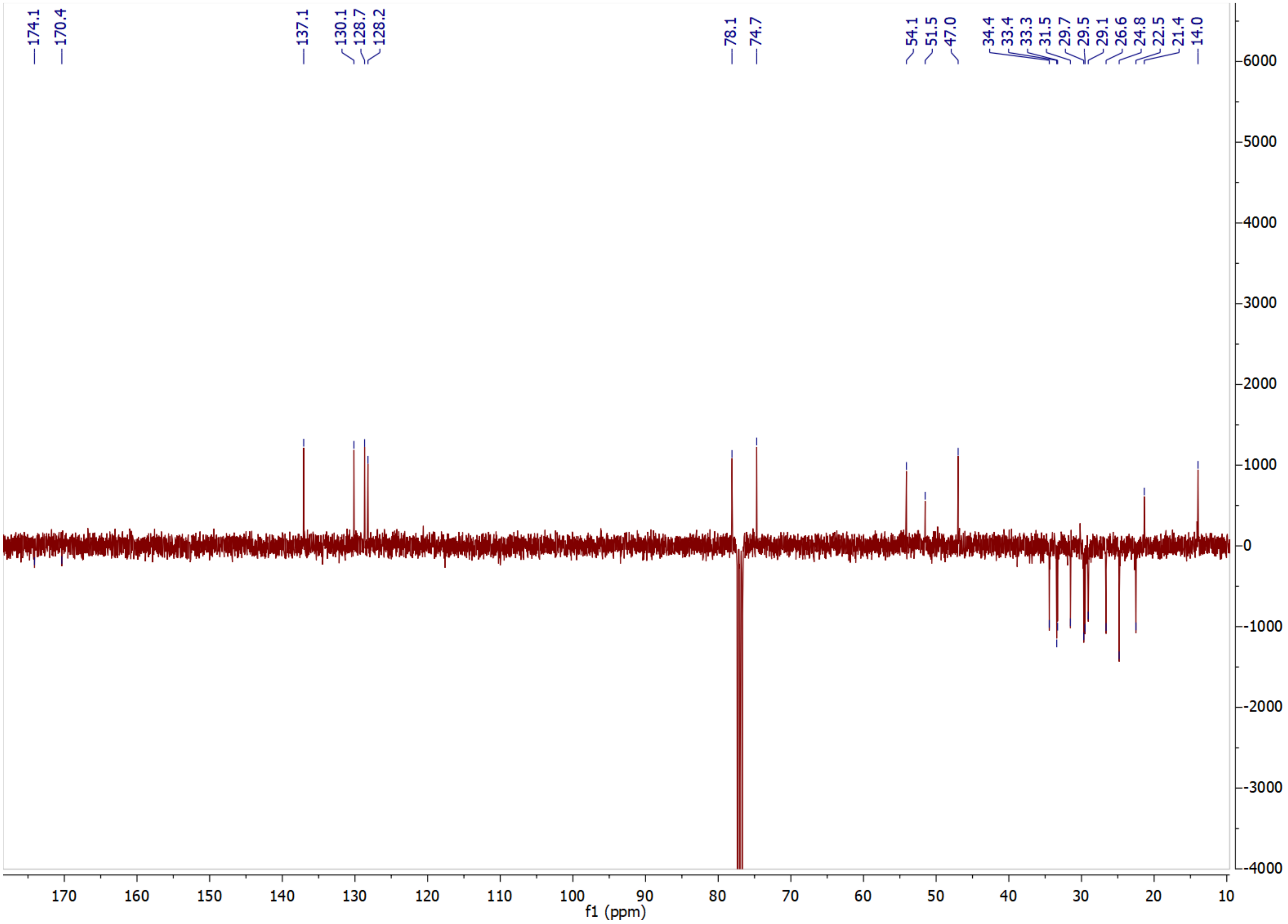

Figure S13: ^{1}H NMR spectrum of derivative **5** in $CDCl_3$

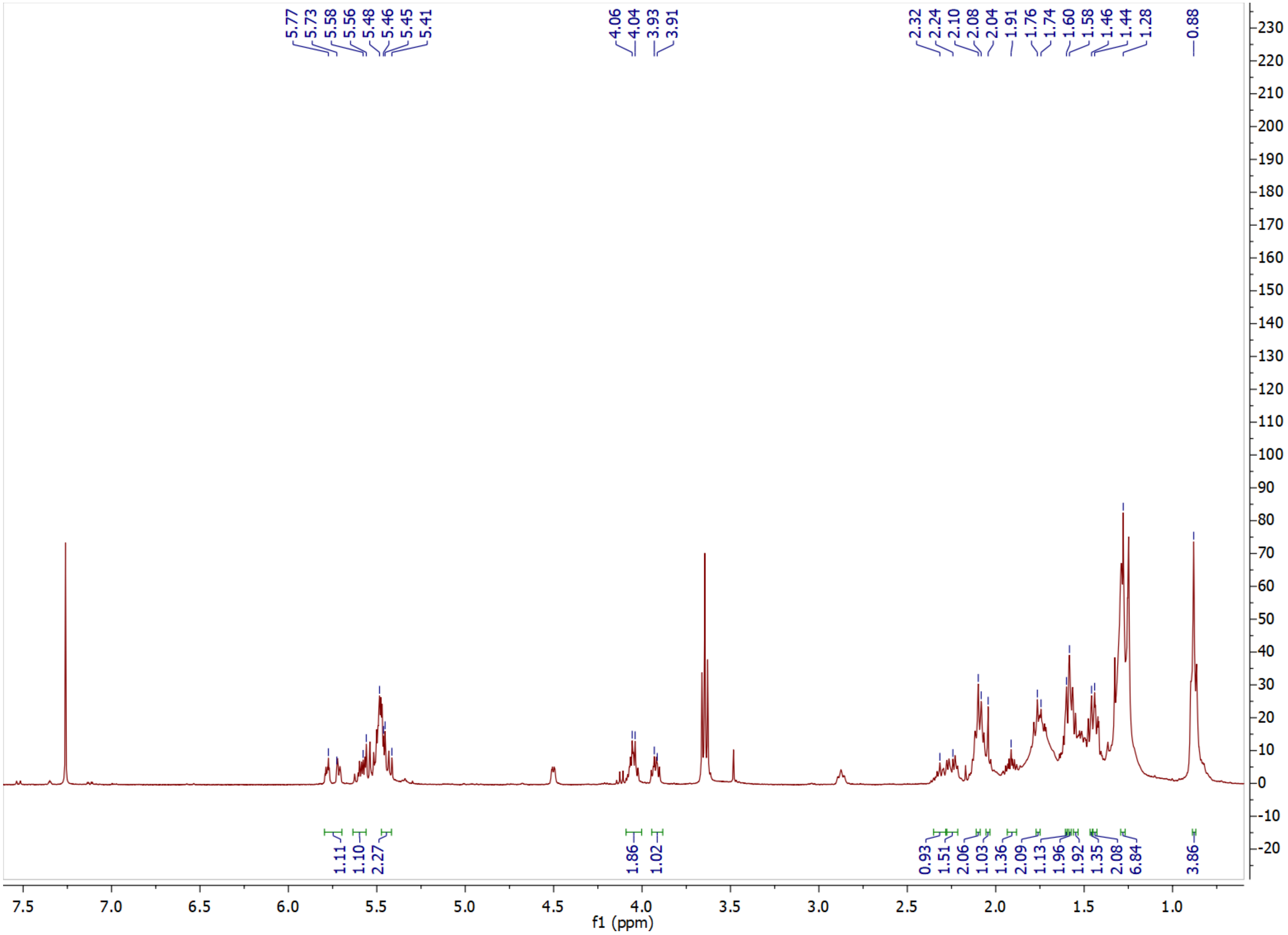

Figure S14: ^{13}C NMR spectrum of derivative **5** in $CDCl_3$

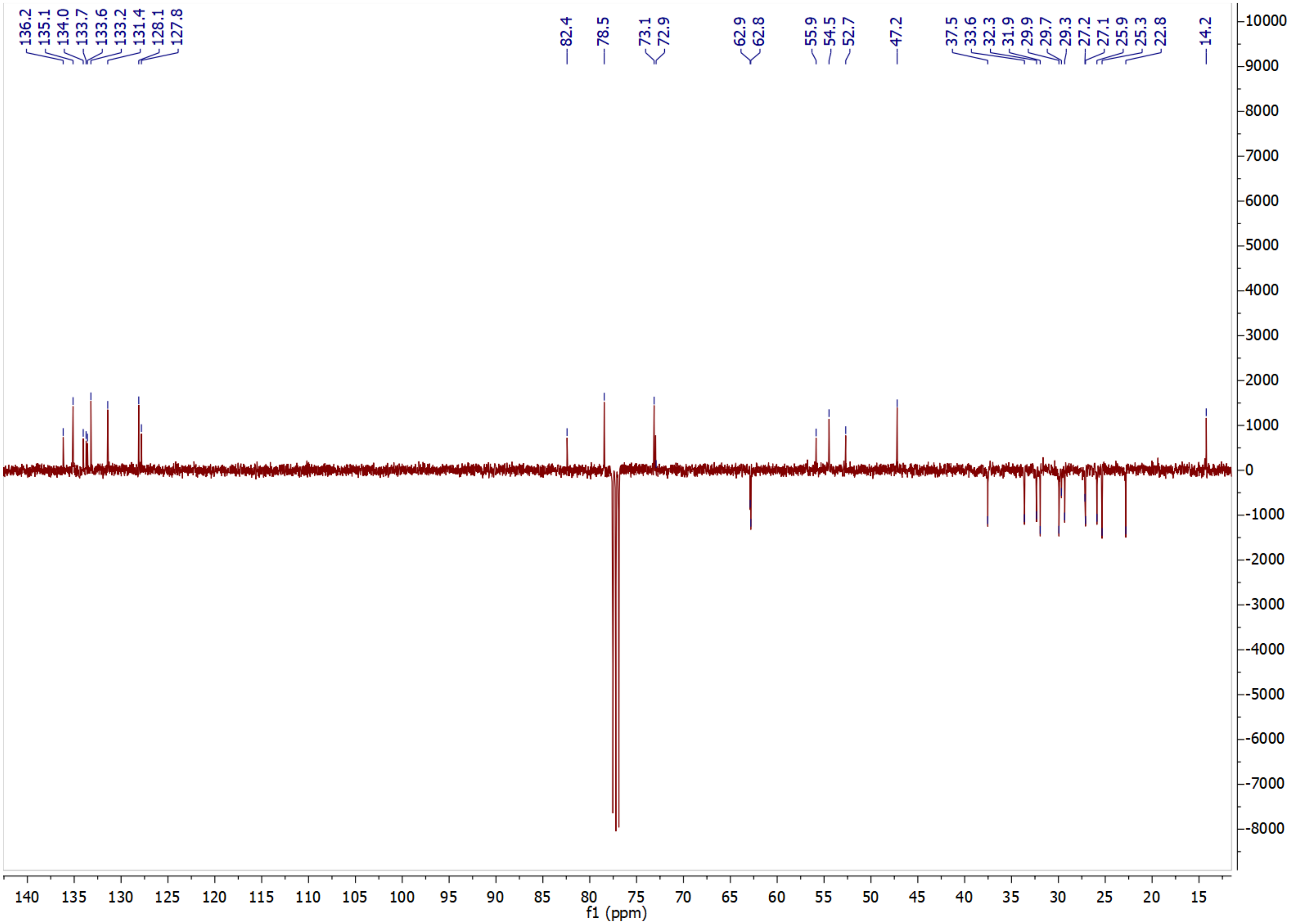

Figure S15: 3D interaction models of test compounds within the active site of p38α-kinase enzyme (PDB ID: 4FA2). a) **1** (blue sticks); b) **2** (light pink sticks); c) **3** (violet sticks); d) **4** (light green sticks); and e) **5** (white sticks). Hydrogen bonds and enzyme residues in dark green lines and light brown sticks, respectively.

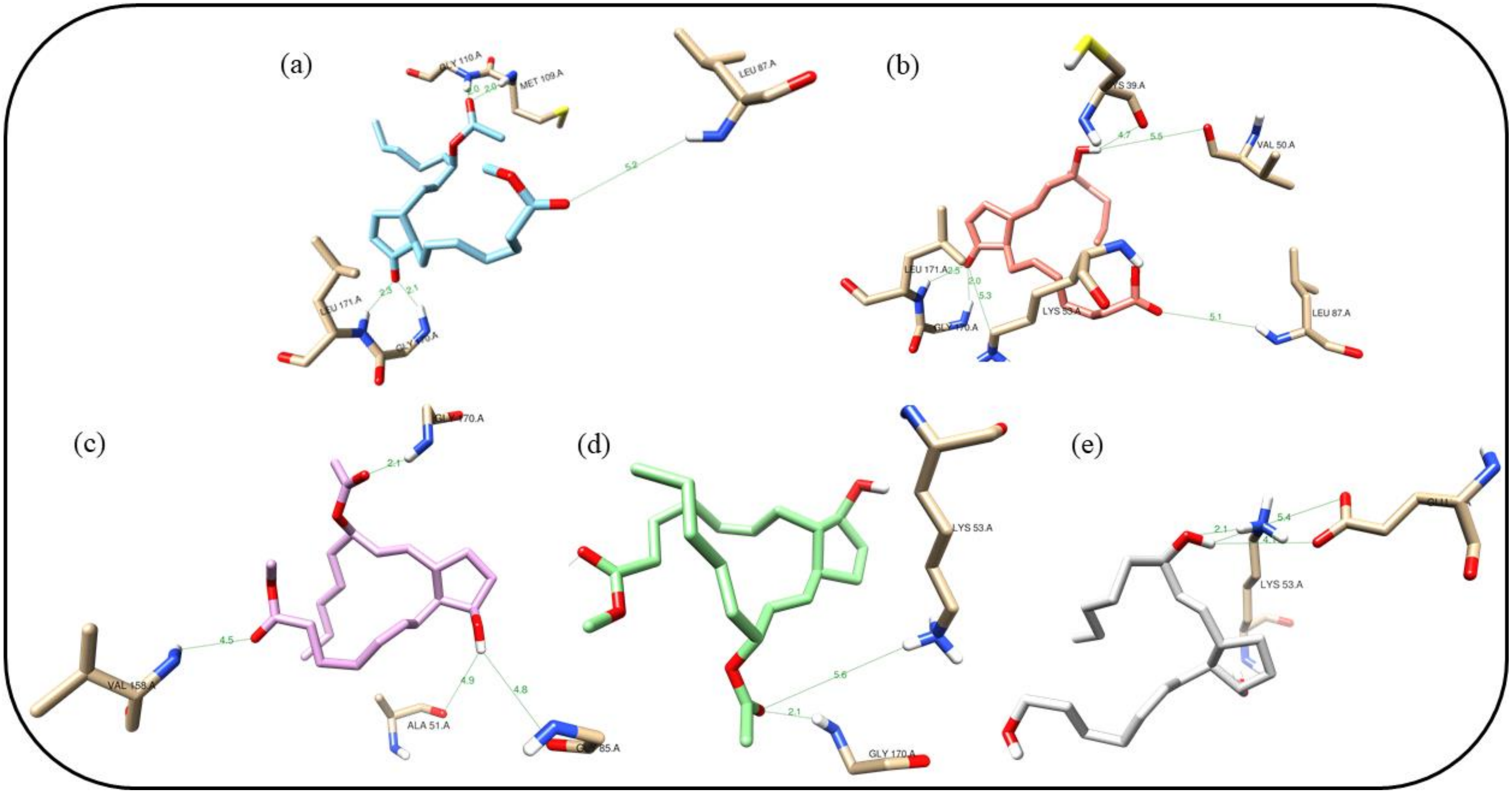

Figure S16: 3D interaction models of test compounds within active site of topoisomerase IIα enzyme (PDB ID: 1ZXM). a) **1** (blue sticks); b) **2** (light pink sticks); c) **3** (violet sticks); d) **4** (light green sticks); and e) **5** (white sticks). Hydrogen bonds and enzyme residues in dark green lines and light brown sticks, respectively.

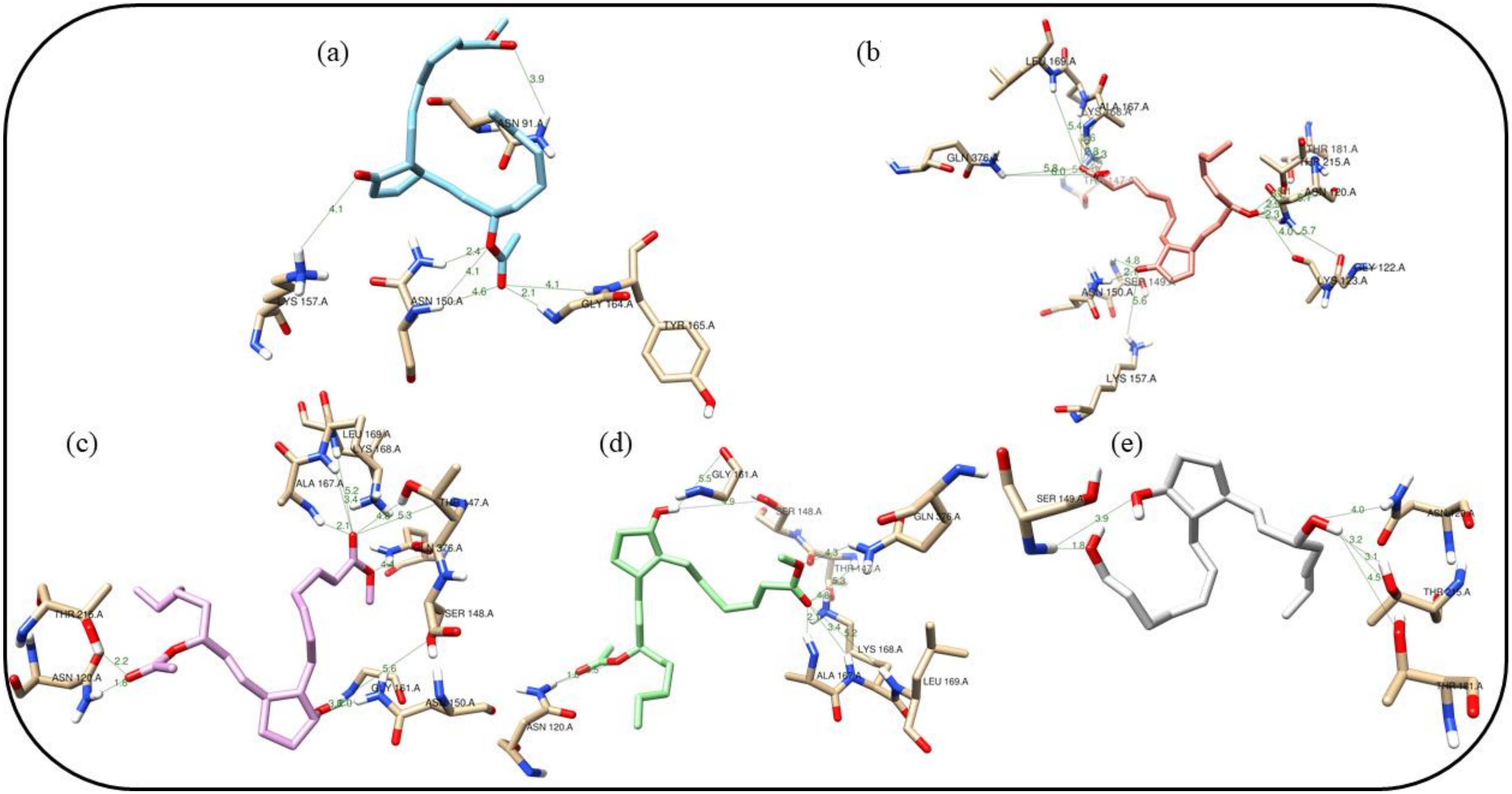

Figure S17: 3D interaction models of test compounds within the active site of Src-kinase enzyme (PDB ID: 2BDF). a) **1** (blue sticks); b) **2** (light pink sticks); c) **3** (violet sticks); d) **4** (light green sticks); and e) **5** (white sticks). Hydrogen bonds and enzyme residues in dark green lines and light brown sticks, respectively.

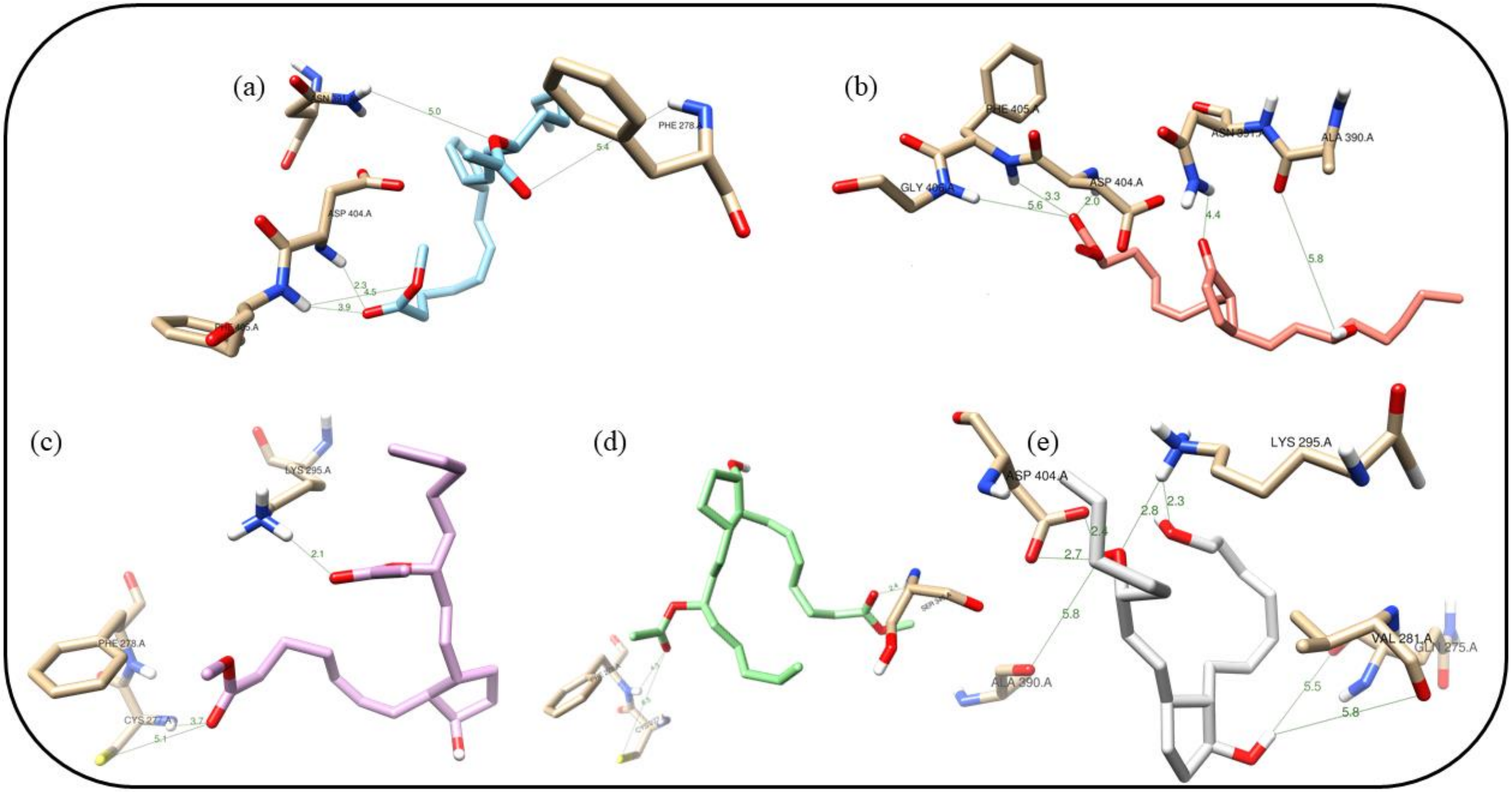

Table S1: Vina scores and binding features for the best pose of each test compound within the active site of p38α-kinase (PDB ID: 4FA2)

Compound	Vina Scores (kcal/mol)	Binding features		
		Residue	Type	Length (Å)
**1**	-8,3	[1]Gly_{110}	*H*-Bond	2.0
		[1]Met_{109}	*H*-Bond	2.0
		[1]Gly_{170}	*H*-Bond	2.1
		[1]Leu_{171}	*H*-Bond	2.3
**2**	-8,0	[1]Gly_{170}	*H*-Bond	2.0
		[1]Leu_{171}	*H*-Bond	2.5
**3**	-8,1	[1]Gly_{170}	*H*-Bond	2.1
**4**	-8,1	[1]Gly_{170}	*H*-Bond	2.1
**5**	-8,5	[3]Lys_{53}	*H*-Bond	2.1

[1]Bonding to amide NH group; [2]Bonding to amide carbonyl group; [3]Bonding to side chain R

Table S2: Vina scores and binding features for the best pose of each test compound within the active site of topoisomerase IIα (PDB ID: 1ZXM)

Compound	Vina Scores (kcal/mol)	Binding features		
		Residue	Type	Length (Å)
**1**	-8,2	[1]Gly164	*H*-Bond	2.1
		[3]Asn150	*H*-Bond	2.4
**2**	-8,7	[3]Asn150	*H*-Bond	2.1
		[3]Asn120	*H*-Bond	2.3
		[1]Ala167	*H*-Bond	2.3
		[1]Ala167	*H*-Bond	2.3
**3**	-8,5	[3]Asn120	*H*-Bond	1.8
		[3]Asn150	*H*-Bond	2.0
		[1]Ala167	*H*-Bond	2.1
		[3]Thr215	*H*-Bond	2.2
**4**	-8,6	[3]Asn120	*H*-Bond	1.8
		[1]Ala167	*H*-Bond	2.1
**5**	-8,0	[1]Ser149	*H*-Bond	1.8

[1]Bonding to amide NH group; [2]Bonding to amide carbonyl group; [3]Bonding to side chain R

Table S3: Vina scores and binding features for the best pose of each test compound within the active site of Src-kinase (PDB ID: 2BDF)

Compound	Vina scores (kcal/mol)	Binding features		
		Residue	Type	Length (Å)
**1**	-8,3	[1]Asp404	*H*-Bond	2.3
**2**	-8,9	[1]Asp404	*H*-Bond	2.0
		[3]Glu310	*H*-Bond	2.2
**3**	-7,9	[3]Lys295	*H*-Bond	2.1
**4**	-8,1	[1]Ser345	*H*-Bond	2.4
**5**	-8,1	[3]Lys295	*H*-Bond	2.3
		[2]Asp404	*H*-Bond	2.4
		[3]Asp404	*H*-Bond	2.7

[1]Bonding to amide NH group; [2]Bonding to amide carbonyl group; [3]Bonding to side chain R
